# Advancements in RNA-based therapies from bench to bedside

**DOI:** 10.1038/s44386-025-00037-y

**Published:** 2026-02-09

**Authors:** Amrita Singh, Muktika Tekade, Sreeharsha Nagaraja, Azad Bharti, Rakesh Kumar Tekade

**Affiliations:** 1 National Institute of Pharmaceutical Education and Research (NIPER) Ahmedabad, Department of Pharmaceuticals, Ministry of Chemicals and Fertilizers, Government of India, Gandhinagar, India; 2 School of Pharmaceutical Sciences, Lovely Professional University, Phagwara, India; 3 Department of Pharmaceutical Sciences, College of Clinical Pharmacy, King Faisal University, Al-Ahsa, Saudi Arabia

**Keywords:** Biological techniques, Biotechnology, Drug discovery

## Abstract

RNA research has advanced rapidly, becoming a key component of modern therapeutics. This review outlines key milestones and innovations from antisense oligonucleotides and RNA interference to mRNA therapies and aptamers that have enabled targeted treatments for diverse diseases. The success of COVID-19 mRNA vaccines highlighted the remarkable clinical potential of RNA-based technologies. Emphasis is placed on delivery technologies, landmark approvals, and ongoing trials shaping the future of RNA-based medicine.

## Introduction

RNA therapy refers to a therapeutic approach that utilizes ribonucleic acid (RNA) molecules to treat diseases. It involves the use of various types of RNA molecules, such as messenger RNA (mRNA), small interfering RNA (siRNA), microRNA (miRNA), and antisense RNA, to regulate gene expression, protein production, or other cellular processes involved in disease pathology. RNA therapy can be used to target a wide range of conditions, including genetic disorders, infectious diseases, cancer, and autoimmune disorders. The primary goal of RNA therapy is to modulate gene expression or protein activity, thereby correcting aberrant cellular functions and restoring health.

RNA-based therapies represent a promising class of treatments that harness the cellular machinery to address various diseases at the genetic level. RNA-based therapies hold great promise for revolutionizing disease treatment by offering more effective, precise, and personalized interventions. These therapies enable the accurate targeting of disease-causing genes or proteins, offer versatility across various disease mechanisms, and facilitate rapid development compared to traditional drugs. With reduced immunogenicity compared to viral vectors, these novel therapeutic approaches foster innovation and advancement in medicine. In this review, the term ‘RNA therapeutics’ refers to agents in which RNA molecules function as the therapeutic entity itself (e.g., siRNA, miRNA, mRNA vaccines, antisense oligonucleotides (ASOs), or aptamers), whereas ‘RNA-based therapeutics’ includes both RNA therapeutics and RNA-dependent technologies such as CRISPR–Cas systems and RNA-guided editing tools. This distinction is important for delineating direct RNA drug modalities from RNA-utilizing therapeutic platforms^1^. In recent years, RNA-based therapies have emerged as a groundbreaking approach in medicine, offering innovative solutions to various diseases by leveraging the inherent capabilities of RNA molecules within the human body.

### Historical evolution of RNA-based therapeutics

RNA therapy is now a vital tool for treating human diseases, as evidenced by several recent discoveries. When Crick first described RNA in his groundbreaking paper, “Central Dogma of Molecular Biology,” it became clear that RNA was essential to transmitting genetic information^2^. The subsequent identification of mRNA provided conclusive evidence, further underscoring the critical role of these molecules as essential messengers in genetic information translation^3,4^.

Another significant, yet less discussed, breakthrough in the realm of RNA research was the discovery that two RNA molecules form base pairs with each other. This discovery, now commonly accepted, challenged early assumptions that RNA could not adopt a double helix structure. In 1956, Rich and Davies published pioneering work on nucleic acid hybridization reactions, demonstrating that RNA could indeed form a structure akin to DNA through complementary base pairing^5^. This seminal finding laid the groundwork for subsequent discoveries, such as microRNAs in 1993 and RNA interference in 1998. In both cases, the formation of RNA duplexes plays a crucial role in RNA silencing mechanisms^6,7^.

The first use of RNA base pairing for therapeutic purposes was described by Stephenson and Zamecnik in 1978. They created an ASO that was intended to target the Rous sarcoma virus (RSV) 35S RNA sequence to hinder viral multiplication^8^. This pioneering work laid the foundation for RNA-based therapeutics. Nearly two decades later, the United States Food and Drug Administration (US FDA) sanctioned the first drug employing an ASO for the treatment of cytomegalovirus retinitis^9^.

RNA splicing, a post-transcriptional process that connects exons and eliminates introns from primary RNA transcripts, was initially discovered in 1977^10^. It is now recognized as a key mechanism in disease, with alterations in splicing being associated with numerous human disorders^10–12^. While traditionally considered challenging with conventional small-molecule drugs, modulation of splicing defects has now been shown to be feasible through both RNA-targeting small molecules and RNA-based therapeutics. A notable example is risdiplam, a small-molecule splicing modulator approved by the FDA in 2020 that promotes the inclusion of exon 7 in *SMN2* transcripts to treat spinal muscular atrophy (SMA)^13^.

In parallel, ASOs such as nusinersen and eteplirsen have demonstrated that targeted manipulation of pre-mRNA splicing can correct disease-associated defects at the transcript level^14^. The first experimental evidence of antisense-mediated splicing correction was reported by Dominski and Kole (1993), who restored β-globin splicing in a β-thalassemia model, establishing the conceptual foundation for modern splice-modulating RNA therapies^15^. Correcting or adjusting splicing defects is nearly impossible with traditional small-molecule drugs, but it can be achieved using RNA-based therapies, particularly ASOs. A 1993 study was the first to demonstrate that alternative splicing could be influenced through the use of ASOs^15^. In this research, a group of ASOs was employed to target the splice sites and branch points of thalassaemic pre-mRNA, aiming to correct abnormal splicing and alleviate symptoms. This work now stands as a foundational reference in advancing new treatments for various challenging neurological disorders^16^.

Compared to the prolonged evolution of antisense oligo-based drugs, the transition from the discovery of small interfering RNAs to their practical use as therapeutic medications occurred relatively quickly. The concept of RNA interference was initially elucidated in a groundbreaking paper in 1998, demonstrating that the administration of sense and antisense RNAs to *Caenorhabditis elegans* embryos effectively and specifically inhibited targeted endogenous mRNAs^7^. Due to its simplicity and efficacy, RNAi gained rapid acceptance within the scientific community and saw extensive application in a remarkably short period. An illustrative example is a study from 2002 that showcased the use of RNAi to suppress hepatitis C virus replication in mice, leading to widespread exploration of RNAi for therapeutic purposes^16^. This impetus led to the first RNAi technology-based clinical trials, which began in 2010, when a patient with extensive melanoma received a siRNA targeting the M2 subunit of ribonucleotide reductase.

Notably, this trial demonstrated the successful cleavage of the target mRNA when the siRNA was delivered utilizing a targeted nanoparticle delivery system^17^. Following this significant achievement, other siRNA-based medications were evaluated for various illnesses; in 2018, the first siRNA medication was licensed for use in patients with hereditary transthyretin-mediated amyloidosis^18^. Despite mRNA being identified as a genetic translation messenger in 1961, it took nearly three decades for researchers to harness it for therapeutic purposes^19^. The identification of RNA-related enzymes, like RNA-dependent RNA polymerase (also called RNA replicase) and reverse transcriptase, made decades ago, has played a crucial role in the progress of today’s mRNA-based therapies^20^.

In the 1990s, efforts to generate specific proteins by introducing exogenous mRNA began, marking a pivotal chapter in the field of scientific exploration. Wolff and colleagues’ groundbreaking work in 1990, where they injected mRNAs for reporter genes directly into mouse skeletal muscle, laid the foundation for utilizing exogenous mRNA to express specific proteins in vivo^21^. This early success paved the way for exploring mRNA as a potential tool for vaccination. In 1993, mRNA transcripts were first evaluated as a vaccine when researchers synthesized mRNAs for the influenza nucleoprotein and encapsulated them into liposomes for injection into mice^22^. This study demonstrated the ability of mRNA vaccines to induce an immune response, specifically the production of virus-specific cytotoxic T lymphocytes. The potential of mRNA vaccines in cancer treatment was realized by Conry and colleagues in 1995 when they designed the first mRNA vaccine targeting human carcinoembryonic antigen (CEA)^23^. When injected safely into CEA-expressing tumor cells, this vaccine showed promising results in mouse models. Although initial progress was promising, it took several years for mRNA-based therapies to reach human clinical trials. It was not until 2008 that Weide and collaborators published the first clinical trial outcomes involving mRNA vaccines in patients with metastatic melanoma^24^. Protamine-protected mRNAs encoding tumor-associated antigens were used in these vaccines, and the outcomes demonstrated an increase in vaccine-directed T cells, suggesting a possible therapeutic benefit^25^. The journey from early animal experiments to human clinical trials highlights the gradual but significant progress made in harnessing the potential of mRNA for therapeutic purposes.

**Fig. 1:**
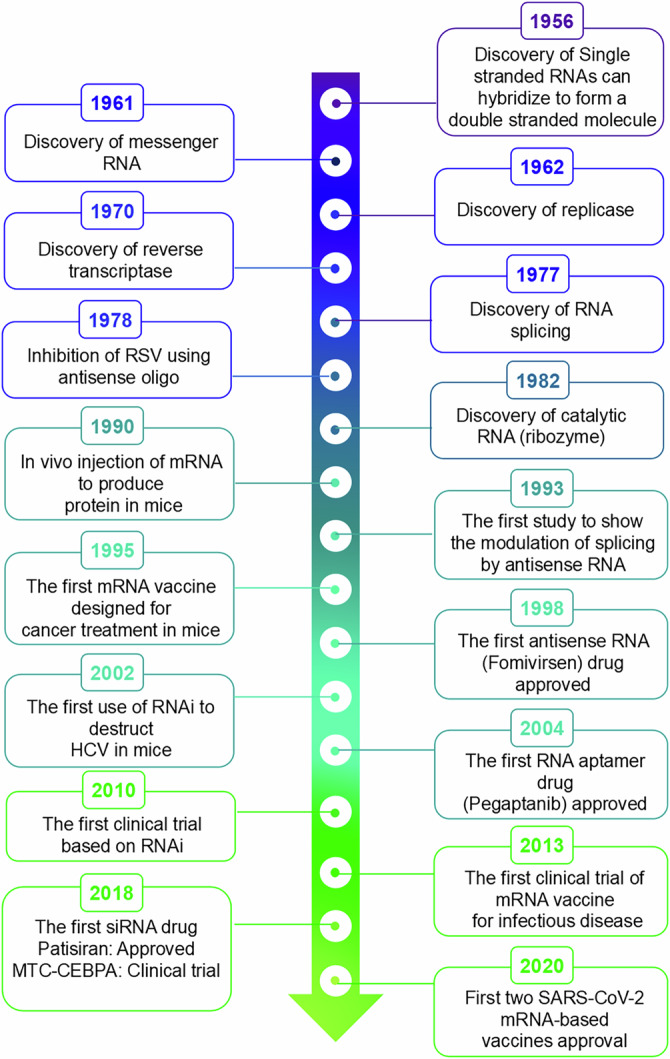
The historical timeline of essential discoveries in RNA biology and key developments in RNA therapy. Timeline highlighting major milestones in the understanding of RNA biology and the development of RNA-based therapeutic technologies.

The journey of mRNA vaccines in combating infectious diseases began with a landmark clinical trial in 2013 (NCT02241135). This trial investigated a novel rabies vaccine that harnesses mRNA technology to deliver its glycoprotein. Results were promising, demonstrating the ability of the vaccine to stimulate the production of effective antibodies against the targeted viral antigens, marking a pivotal advancement in mRNA-based vaccine development^26^. 2020 marked a significant milestone in medical history with the approval of the first mRNA-based vaccination against the coronavirus that causes severe acute respiratory syndrome (SARS-CoV-2)^26^. This brief description, embraced by nations worldwide, represents the culmination of over four decades of dedicated research and underscores the transformative potential of mRNA therapeutics in shaping the future of global healthcare. This concise overview highlights the extensive legacy of RNA-based therapeutics, with Fig. 1 summarizing the key historical milestones in RNA biology and therapeutic development.

## RNA-based therapeutics and their biomechanics

Long-term research into various RNA types has led to a deeper understanding of cellular mechanisms and the rapid development of RNA-based therapies. These therapies, including ASOs, small interfering RNAs, microRNAs, and modified messenger RNAs, hold promise for modulating gene expression and treating genetic diseases^27^. RNA therapeutics can be broadly categorized into four main domains: antisense technologies, mRNA-based strategies, RNA interference-based therapeutics, and CRISPR–Cas-mediated genome editing. Each approach offers unique ways to treat diseases by targeting specific genes or genetic processes.

### Antisense technologies

The year 1978 marked the introduction of the concept of creating oligonucleotides to bind to particular sequences within target RNAs through Watson-Crick hydrogen bonding. This concept and the term “antisense” initially represented a straightforward idea, although it lacked specificity. Double-stranded RNA was discovered by Andrew Fire and Craig Mello in 1998 to be the cause of RNA interference in *Caenorhabditis elegans*^7^. The authors did not outline specific mechanisms by which RNA binding modifies the behavior and effectiveness of the targeted RNA. Furthermore, they did not specify whether the administered drug would consist of a single or double strand, nor did they delineate the necessary chemical modifications to ensure therapeutic efficacy.

Antisense refers to any oligonucleotide, irrespective of its composition or chemical makeup, engineered to interact with target RNA through Watson-Crick hybridization. Nearly 50 years ago, Paul Zamecnik and Mary Stephenson created a synthetic ASO in 1978 with the goal of blocking the reproduction of the Rous sarcoma virus in tissue culture. This marked the beginning of the development of antisense-based treatments^28^. These investigations introduced the concept of leveraging the distinctive chemical attributes of nucleic acids in pharmaceutical development. Initially, this concept and the term “antisense” seemed straightforward, albeit lacking specificity. The US FDA did not approve the first ASO medication for clinical use until two decades later^27^.

**Fig. 2:**
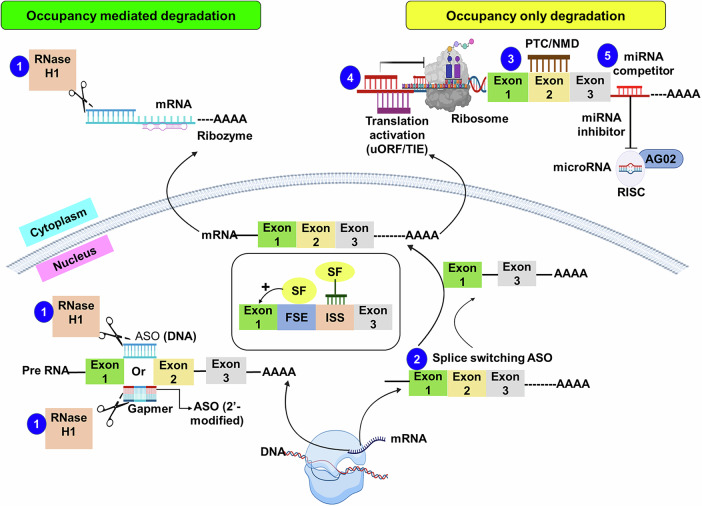
Antisense oligonucleotides (ASOs) mediated gene regulation. There are two ways that ASOs can alter the expression of a target gene. (1) In the occupancy-mediated degradation pathway, ASOs cause ribozymes or RNase H1 to cleave the target mRNA. It is not the case that the occupancy-only mechanisms directly damage target RNA. Instead, it controls the expression of genes in multiple ways: (2) causes nonsense-mediated mRNA decay (NMD); (3) inhibits or activates translation; (4) changes RNA splicing by employing splice-switching ASOs to induce exon skipping or exon inclusion; (5) impedes the microRNAs binding to target mRNA (Created with BioRender.com).

Synthetic 21-nucleotide (nt) ASO fomivirsen inhibits the synthesis of proteins essential for CMV replication by interacting with a complementary mRNA region of the virus^29^. With its approval, it was intended to treat CMV retinitis, a severe infection of the retina that is common in people with acquired immune deficiency syndrome (AIDS) and can result in blindness^30,31^. The medicine was taken off the market despite its therapeutic benefits because anti-retroviral therapy was so successful. Nevertheless, the first demonstration of the clinical utility of ASOs was given by fomivirsen, which provided first clinical proof-of-concept for ASO despite later market withdrawal due to changing clinical practice More recent approvals (and ongoing trials, given in Table 1) highlight the importance of route-of-administration and tissue targeting for efficacy and safety: intrathecal delivery enabled CNS exposure for nusinersen, whereas systemic ASOs require careful chemistry to balance stability, protein binding, and off-target effects. Together, these trials emphasize that ASO success depends as much on delivery and patient selection as on target biology^32^. Progress in RNA biology has facilitated the advancement of ASOs that operate through various mechanisms after binding to RNA. These mechanisms can generally be categorized into two groups based on different post-hybridization mechanisms: those involving the degradation of target RNAs mediated by occupancy (via cleavage) and those involving only occupancy (through steric interference)^33,34^. ASO-mediated mechanism delineated in Fig. 2.

**Table 1:** Antisense oligonucleotide drugs with either FDA approval or in clinical trials

Drug/alternative name	Target organ	Dose/route	Indication	Key observation	Organization	Updated status	References
Fomivirsen	Eye	Intravitreal	Cytomegalovirus retinitis, HIV infections	The first FDA-approved ASO medication (1998), withdrawal in Europe and the USA in 2002	Ionis Pharmaceuticals	Completed NCT00002187, NCT00002355	^230^
Mipomersen/Kynamro, ISIS 301012	Liver	Subcutaneous	Hypercholesterolemia, atherosclerosis, coronary artery disease	The second generation of ASO (Gapmer ASO)	Kastle and Ionis Therapeutics	Completed NCT01598948 NCT00607373 NCT01475825 NCT00770146 NCT00694109 NCT00794664	^231^
Inotersen/ISIS 420915, Tegsedi, AKCEA- TTR-LRx	Liver	Subcutaneous	Hereditary ATTR amyloidosis (hATTR)	--	Ionis Pharmaceuticals	Completed NCT01737398	
Nusinersen/ISIS 396443, Sprinraza, IONIS- SMN Rx, BIIB058	Central nervous system	Intrathecal	Spinal muscular atrophy (SMA)	Approved in 2016	Ionis Pharmaceuticals and Biogen	Completed NCT02292537	^231^
Eteplirsen/AVI-4658, EXONDYS 51	Muscle	Intravenous	Duchenne muscular dystrophy (DMD)	The third generation of ASO medication	Sarepta Therapeutics	Completed NCT02255552	^232^
Golodirsen/SRP-4053,Vyondys53	Muscle	Intravenous	Duchenne muscular dystrophy (DMD)	With advanced chemical modifications	Sarepta Therapeutics	Recruiting NCT02500381	^233^
Casimersen/SRP-4045, Amondys, 45	Muscle	Intravenous	Duchenne muscular dystrophy (DMD)	--	Sarepta Therapeutics, Inc.	Recruiting NCT03532542 NCT02500381	^234^

#### Occupancy-mediated degradation

Occupancy-mediated degradation involves the precise binding of ASOs to specific RNA sequences, leading to the cleavage of RNA at the ASO binding sites by endogenous enzymes^35^. Occupancy-mediated degradation of target RNA has demonstrated effectiveness as a productive approach, with several mechanistic strategies validated for its implementation. The most thoroughly characterized mechanism is RNase H1-mediated degradation, in which RNase H1 functions as a highly selective endonuclease, cleaving RNA within double-stranded RNA: DNA hybrids. Subsequently, the RNA fragments from this process are degraded by 5’ and 3’ exonucleases. RNase H1 requires a substrate containing 8–10 consecutive ribonucleotide-containing base pairs (bp) for optimal activity. Consequently, ASOs that facilitate RNase H1-mediated degradation of the target RNA fragments typically possess a central segment composed of eight to ten deoxynucleotides, flanked by multiple 2ʹ-modified nucleotides, known as chimeric or gapmer ASOs. This structural configuration enhances the affinity of ASOs for their target RNA and increases their resistance to nucleases, thereby prolonging their activity duration. Due to its widespread presence, RNase H1 effectively targets both cytoplasmic and nuclear transcripts^36^. Other enzymes, such as ribozymes, also contribute to degradation through occupancy-based mechanisms^37^. Ribozymes cleave target RNAs using structural motifs, such as hammerhead or hairpin formations. Moreover, their substrate recognition domains can be modified to enable precise, site-specific cleavage in either a *cis* or *trans* manner^38^.

#### Occupancy-only mechanisms

In occupancy-only mechanisms, ASOs regulate the up- or downregulation of target transcripts solely through binding, independent of specific enzymes. In the occupancy-only or steric-blocking, mechanism of action, ASOs regulate RNA processing and function without inducing RNase H1-mediated cleavage^39^. Instead, they act by physically blocking the access of cellular machinery to specific RNA sequences. First, ASOs can bind to pre-mRNA at splice-junctions or splicing-regulatory elements, thereby preventing spliceosome assembly or altering exon recognition, resulting in modulation of exon inclusion or exclusion^35^. This steric interference enables the correction of aberrant splicing, as exemplified by *nusinersen*, which restores exon 7 inclusion in *SMN2* transcripts to treat spinal muscular atrophy^40^. Second, ASOs may hybridize to the 5′ untranslated region (UTR) or start codon region of mRNA, blocking ribosomal recruitment and translation initiation. Third, ASOs can interfere with RNA–protein or RNA–microRNA interactions, modifying RNA stability, nuclear export, or localization. Collectively, these occupancy-only mechanisms enable RNA modulation without transcript degradation, broadening therapeutic applications for targets not amenable to RNase H1–dependent strategies^41,42^.

Clinical trials have examined translation-blocking ASOs, including those designed to inhibit MYC translation, as a potential treatment for cancer^43^. ASOs can also direct the cleavage of 5’ cap structures, thereby blocking translation^44^. ASOs can attach to pre-mRNA sequences associated with cleavage and polyadenylation, influencing the selection of polyadenylation sites and consequently modifying mRNA stability and levels^45^. The production of desirable mRNAs and their encoded proteins can be enhanced through various mechanisms.

To date, the most widely used method in developing agents that have entered clinical trials involves designing ASOs that modify splicing, such as Phosphorothioate (PS) ASOs and phosphorodiamidate morpholino oligomers^46–49^. A prime example is the action mechanism of nusinersen, a recognized ASO used to treat spinal muscular atrophy (SMA). This condition results from the homozygous loss of function of the SMN1 gene, which encodes the SMN protein, crucial for developing and maintaining neuromuscular junctions. Humans also have a backup SMN2 gene that can produce small amounts of full-length SMN. However, this gene carries a mutation in a splicing enhancer sequence, causing exon 7 to be skipped, leading to a shorter, unstable SMN variant. Nusinersen, a phosphorothioate (PS) ASO, targets SMN2 pre-mRNA, enhancing the inclusion of exon seven and allowing the production of the full-length protein in motor neurons within the central nervous system (CNS). This approach provides a highly effective treatment for SMA^14,50^.

Additionally, ASOs can influence precursor mRNA by modulating the splicing process. This is accomplished either by directly obstructing splice junctions or by inhibiting the binding of splicing regulatory proteins that promote or suppress splicing^48,51^. Occupancy-only ASOs can also activate the natural cell surveillance pathways that remove defective mRNAs^52^. In another mechanism, changes in splicing patterns can lead to the formation of mRNAs with premature stop codons, which are selectively degraded via the nonsense-mediated mRNA decay (NMD) pathway^52^. ASOs can also interfere with mRNA translation by directly obstructing the process. This interference can activate the no-go decay pathway, a cellular quality control system that identifies and degrades mRNAs where ribosomes become stalled^53^.

Overall, the discovery of new mechanisms of action enhances the versatility of antisense technology. Having multiple pathways to degrade target RNAs enables effective reduction of RNA levels, even for those that are less susceptible to RNase H1-mediated cleavage, such as RNAs with very short half-lives or high translation rates^54^. Integrating mechanisms that selectively boost protein translation indicates that this technology can now do more than simply promote alternative splicing in individuals with loss-of-function mutations.

#### Limitations

Delivering ASOs in clinical applications presents several challenges, including vulnerability to degradation by serum nucleases, rapid renal clearance, limited tissue penetration, and low cellular uptake. The widely used phosphorothioate backbone improves binding with serum proteins, helping to decrease renal clearance^55^. Additionally, chemical modifications enhance cellular uptake by engaging surface receptors that facilitate oligonucleotide endocytosis. However, once inside the cell, ASOs need to escape the endosome to exert their activity, posing an additional challenge^56^.

Another challenge facing ASO therapeutics lies in their tendency to provoke immune responses. The human immune system is adept at identifying single-stranded and double-stranded RNA through pattern-recognition receptors (PRRs)^57,58^. Recognition of RNA occurs extracellularly via endosomal Toll-like receptors (TLRs), particularly TLR-3, 7, and 8^59^. Cytoplasmic defense systems, including protein kinase R (PKR), oligoadenylate synthases (OASes), and RIG-I-like receptors, are responsible for detecting intracellular pathogens^60^. These pathways can be activated, leading to RNA degradation, disruption of cellular translation, and inflammatory responses. Synthetic RNA therapies commonly incorporate 2′-ribose and base modifications such as 2′-O-methyl, 2′-O-methoxyethyl, and 5-methylcytosine (m⁵C), to enhance nuclease resistance, reduce immunogenicity, and improve pharmacokinetic stability^35,61^.

Advancements in understanding the molecular processes that govern the efficacy, spread, cellular absorption, and adverse effects of ASOs provide a foundation for tailoring this adaptable technology to various ailments. Even though all ASO medications currently sanctioned are designated for individuals with uncommon illnesses, innumerable ASOs undergoing clinical trials aim to address prevalent conditions like cardiovascular diseases, metabolic disorders, and cancer. Although achieving broad application encounters obstacles, such as delivering ASOs to specific cell types and the risk of adverse effects with prolonged administration, ASO treatments are anticipated to significantly impact numerous diseases that currently lack effective treatment options.

### RNAi-based therapeutics

**Fig. 3:**
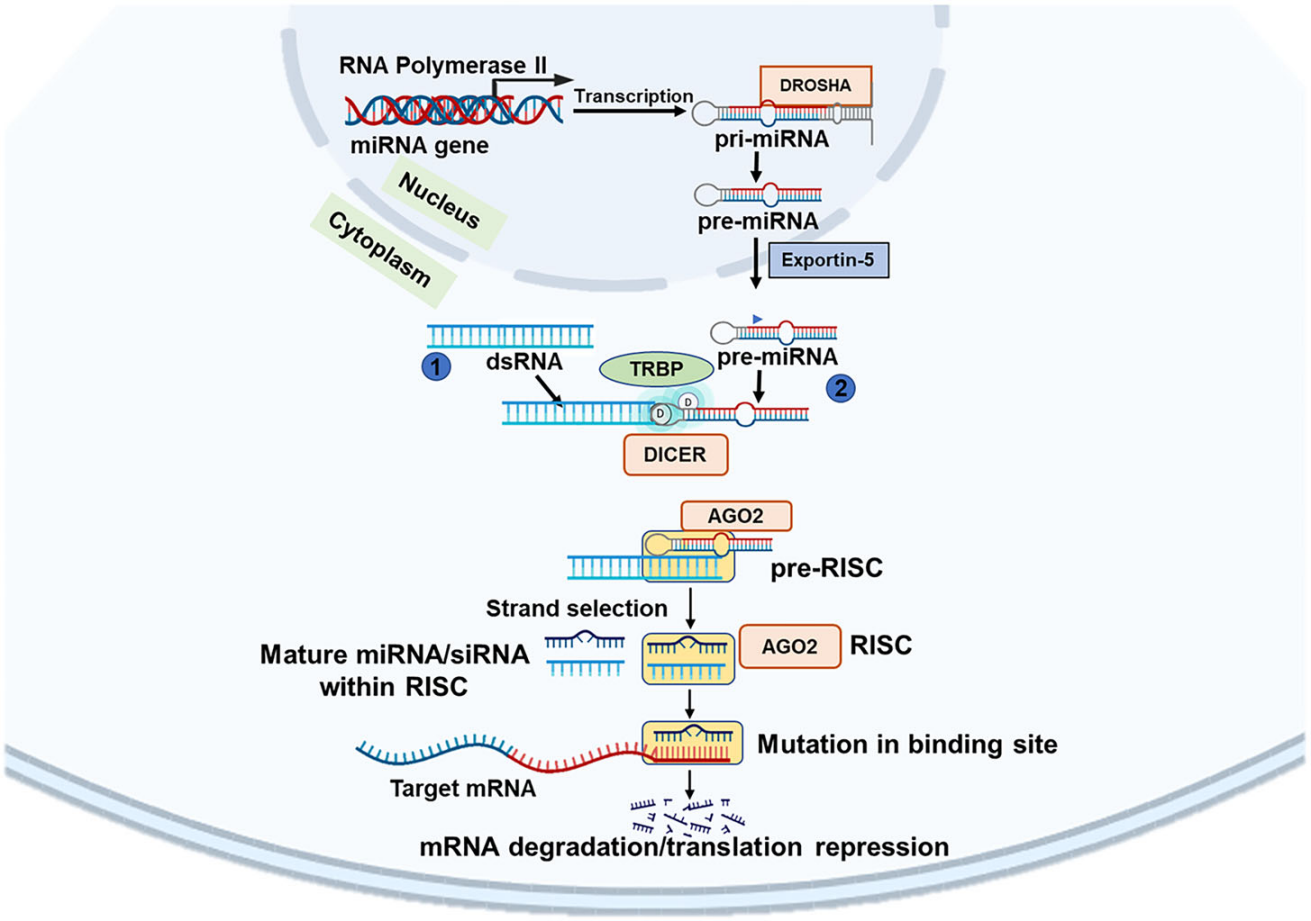
RNA interference (RNAi) regulates gene expression. Long double-stranded RNA (dsRNA) and precursor microRNA (pre-miRNA) are processed by Dicer into short interfering RNA (siRNA). The antisense strand of siRNA (a blue strand) is incorporated into the RNA-induced silencing complex (RISC), which targets RNA for degradation or inhibits translation (Created with BioRender.com).

In *Caenorhabditis elegans*, double-stranded RNA initiates RNA interference, as Andrew Fire and Craig Mello determined in 1998^7^. RNAi, a natural cellular process, initiates the breakdown of particular RNA targets upon detecting double-stranded (ds) RNAs. This process acts as an inherent defense mechanism against invading viruses and transposable elements^27^. Their findings challenged the prevailing belief that antisense RNAs function by directly binding to inhibit the expression of target mRNA. Consequently, an enzymatic mechanism was revealed. The cytoplasmic RNase III enzyme Dicer processes double-stranded RNAs, whether endogenously produced or externally delivered as precursor RNAs with stem-loop or short hairpin structures, into small interfering RNAs or microRNAs. These small RNAs guide the RNA-induced silencing complex (RISC), a ribonucleoprotein assembly comprising an Argonaute protein and the siRNA or miRNA, to regulate specific target mRNAs. This Argonaute protein, along with other complex components, serves as the effector molecule. Conversely, miRNAs typically engage with partially complementary targets, resulting in translational repression and degradation of transcripts^62^. The RNAi pathway in human cells demonstrates exceptional efficiency due to the ability of RISC to trigger several rounds of RNA cleavage^63^. RNAi-based therapies capitalize on this characteristic, as well as the flexibility and controllability of the RNAi machinery. However, the necessity to involve this machinery also imposes limitations on the design of siRNA drugs. RNAi-mediated gene regulation is represented in Fig. 3.

RNAi has rapidly progressed from concept to clinic with several successful examples that illustrate how delivery chemistry determines clinical use. Patisiran, delivered in lipid nanoparticles (LNPs), produced a clinically meaningful improvement in neuropathy in hereditary transthyretin-mediated amyloidosis (APOLLO trial), establishing intravenous LNP delivery for liver-targeted siRNA. Subsequent GalNAc-conjugated siRNAs (e.g., givosiran for acute hepatic porphyria and inclisiran for PCSK9/LDL-C lowering) demonstrated that receptor-mediated subcutaneous dosing enables potent, durable hepatic silencing with improved dosing convenience. The spectrum of clinical outcomes from systemic IV LNP to subcutaneous GalNAc conjugates highlights a central translational lesson: matching the delivery modality to target organ biology (liver vs extrahepatic) can make the difference between a trial failure and regulatory approval.

Synthetic RNAi triggers are typically made up of perfectly matched double-stranded RNAs that must be split apart so that one strand can serve as a “guide” for loading into RISC. In RNA interference drug design, selecting the appropriate strand is crucial, as only the antisense strand binds to the target mRNA. siRNAs that have a 2-nt 3’ overhang on one side and a blunt end on the other, for example, seem to favor the selection of the strand that has the overhang^64^. Additionally, chemical modifications can also facilitate the loading of the antisense strand^65^. Dicer is required to cleave and transmit RNAi triggers longer than 21 bp to the RISC complex. It is noteworthy that Dicer processing is linked to a more stable choice of the antisense strand, as the RISC guide^66^. Shorter siRNAs avoid the early stages of the RNAi pathway and can be directly inserted into RISC. This has the benefit of reducing the disruption of gene regulation by native miRNAs.

In 2018, the FDA approved the first treatment based on RNA interference-mediated gene silencing. An effective treatment for hereditary transthyretin-mediated amyloidosis (hATTR) is patisiran, a double-stranded small interfering RNA. This is the progressive neurodegeneration triggered by amyloid fibril buildup from misfolded transthyretin protein. Patisiran suppresses transthyretin mRNA in the liver, thereby reducing protein levels in the bloodstream and mitigating amyloid deposition. It comprises two modified 21-mer oligonucleotides enclosed within a lipid nanoparticle optimized for liver cell absorption. Vutrisiran, succeeding patisiran, entered the market in 2022. It operates on the exact RNAi mechanism but leverages advanced stabilization chemistry. This siRNA is linked to N-acetylgalactosamine (GalNAc), enhancing its absorption in liver cells and enabling lower dosage administration^18^. While patisiran requires an intravenous infusion every 3 weeks, vutrisiran treatment involves just one subcutaneous injection every 3 months. The FDA has approved RNAi-based drugs (Table 2), and the extensive array of oligonucleotide drugs in clinical stages suggests RNAi therapeutics will soon find widespread application. For instance, various approaches are under exploration for achieving extrahepatic delivery, including antibody conjugates^67–69^, peptide conjugates^70^, hydrophobic^71^ or lipophilic conjugates^72^, as well as multivalency^73^. Moreover, programmable siRNA pro-drugs, activated in response to specific cellular RNA biomarkers, hold promise for selectively targeting diseased cells over healthy neighboring tissue^74^.

**Table 2:** RNAi-based drugs with either FDA approval or in clinical trials

Drug/alternative name	Target organ	Dose/route	Indication	Key observation	Organization	Updated status	References
Patisiran/ALN-TTR02, ONPATTRO™	Liver	Intravenous	hATTR		Alnylam Pharmaceuticals	NCT03862807, NCT01960348	^18^
Givosiran/ALN-AS1, GIVLAARI	Liver	Subcutaneous	Acute hepatic porphyria	--	Alnylam Pharmaceuticals	NCT03338816	^235^
Lumasiran/ ALN- GO1, OXLUMO	Liver	Subcutaneous	Primary hyperoxaluria type 1 (PH1)	Phae III active; NCT03905694 NCT03681184 NCT04152200	Alnylam Pharmaceuticals	Phae III active; NCT03905694 NCT03681184 NCT04152200	^236^
Inclisiran/ ALN- PCSSC, LEQVIO	Liver	Subcutaneous	Hypercholesterolemia, atherosclerotic cardiovascular disease, renal impairment		Alnylam and Novartis Pharmaceuticals	Phase III completed; NCT03399370 NCT03400800 NCT03397121	^142^
Fitusiran/ALN-AT3SC	Blood	Subcutaneous	Hemophilia A/B		Alnylam Pharmaceuticals and Sanofi Genzyme	Phase III completed; NCT03974113 NCT03417102	^142^
Teprasiran/QPI-1002	Kidney	Intravenous	Cardiac surgery	The first systemically administered siRNA drug to enter human clinical trials	Quark Pharmaceuticals	Phase III completed; NCT02610296	^142^
QPI-1007	Eye	Intravitreal	Primary angle-closure glaucoma	-	Quark Pharmaceuticals	Phase II/III terminated: NCT02341560	^142^

miRNAs play a pivotal role as gene regulators, impacting various physiological processes associated with disease, hence emerging as promising therapeutic targets in this regard^75^. Unlike strict matching, miRNAs require only partial complementarity to recognize their targets. As a result, a single miRNA can bind to many different mRNAs, each with varying levels of affinity. Adjusting or mimicking miRNA activity enables the simultaneous alteration of complex gene expression networks. Although targeting multiple potentially compensatory pathways can be advantageous, it also carries the risk of unintended side effects. miRNA therapeutics are divided into two major parts: miRNA mimics and anti-miRNAs.

#### miRNA mimics

miRNA mimics are synthetic oligonucleotide duplex molecules designed to mimic the function of endogenous miRNAs. When introduced into cells, miRNA mimics can restore or enhance the activity of specific miRNAs that may be deficient or underexpressed in certain diseases. Once inside the cell, miRNA mimics interact with the cellular RNA interference machinery and are incorporated into the RISC. This allows them to regulate gene expression by binding to target mRNAs and inhibiting their translation or promoting their degradation, similar to natural miRNAs. miRNA mimics hold potential for therapeutic intervention in diseases where the dysregulation of specific miRNAs contributes to pathology. They can augment the activity of tumor-suppressive miRNAs in cancer or restore miRNA function in other diseases.

#### Anti-miRNAs

Anti-miRNAs, also known as anti-miRNA oligonucleotides (AMOs), are synthetic molecules designed to inhibit the activity of specific endogenous miRNAs. Anti-miRNAs typically function by complementary base pairing with the miRNA, sequestering it and preventing its interaction with target mRNAs. This can occur through various mechanisms, including steric hindrance, RNase H-mediated degradation, or sequestration into subcellular compartments. Miravirsen, an anti-miRNA therapy, targets miR-122, a liver-specific miRNA crucial for lipid metabolism and the replication of Hepatitis C virus (HCV). By binding to miR-122, miravirsen disrupts its interaction with HCV RNA, hindering viral replication^76^. Despite promising antiviral effects in trials, the rise of potent antiviral treatments for HCV reduced the clinical demand for miravirsen, leading to its recent discontinuation^77^.

#### Limitations

ASOs and siRNA medications have comparable delivery, stability, and immunogenicity issues. Thankfully, siRNA treatments benefit significantly from the advances in backbone, base, and sugar alterations that were first developed for ASO medicines^61^. Furthermore, while duplex siRNAs are larger and more hydrophilic than single-stranded ASOs, their delivery is more complex because their external-facing phosphate groups cause them to be rapidly excreted. In response, scientists have developed targeted ligands and lipid nanoparticles as delivery vehicles, and chemical optimization techniques are improving modified siRNA design to increase the therapeutic utility of RNA interference^78^.

### mRNA therapeutics

The discovery of mRNA-encoded drugs emerged in the 1990s, with the revelation that directly injecting in vitro transcribed (IVT) mRNA into the skeletal muscle of mice resulted in the expression of encoded proteins^21^. Using bacteriophage RNA polymerase, synthetic mRNAs used in therapeutic settings are typically IVT from a DNA plasmid. They have parts like a 5’ cap, a 5’ UTR, an open reading frame (ORF), a 3’ UTR, and a poly(A) tail, and they mimic cellular mRNAs. These components are essential for the stability and translation of mRNA, which in turn affects the success of mRNA therapy. Preclinical investigation of IVT mRNA has accelerated the development of mRNA-based vaccines to fight infectious illnesses and cancer^79^. Modified nucleosides, such as pseudouridine and N1-methylpseudouridine, are frequently incorporated into the mRNA structure to optimize translation. Incorporating these modified nucleosides, especially modified uridine, enhances translation efficiency and shields the IVT mRNA from detection by the innate immune system. This strategy enables higher dosage levels to be administered effectively^80^.

Mechanistically, mRNA vaccines administered through injection enter the cytoplasm of host cells, typically antigen-presenting cells (APCs), where they are translated into specific antigens. These antigens are then presented on the surface of APCs by major histocompatibility complexes (MHCs), triggering both B cell/antibody-mediated humoral immunity and CD4+ T/CD8+ cytotoxic T-cell-mediated immunity^81^. Additionally, injected mRNA encoding immune stimulants like cytokines and chemokines can enhance APC maturation and activation, thereby fostering a T-cell-mediated response and improving the immune landscape within tumor microenvironments^82^. The FDA has approved mRNA vaccines (Table 3), and the extensive array of mRNA in clinical stages suggests these vaccines will soon find widespread application.

**Table 3:** mRNA vaccines with either FDA approval or in clinical trials

Drug/alternative name	Disease	Dose/route	Key observation	Organization	Updated status	References
Tozinameran/BNT-162b2	SARS-COV-2 LNP	Intramuscular	COVID-19 spike glycoprotein modulator	BioNTech SE	Approved in 2021	^237^
Spikevax/mRNA-1273	SARS-COV-2 LNP	Intramuscular	COVID-19 spike glycoprotein modulator	Moderna	Approved in 2022	^84^
mRNA-1273	COVID-19, LNP	Intramuscular	SARS-CoV-2 spikes	Moderna	Clinical trial III NCT04805125	^84^
BNT162b2	COVID-19, LNP	Intramuscular	SARS-CoV-2 spike	Pfizer	Clinical trial IV NCT04961229	^238^
LVRNA009	COVID-19, LNP	Intramuscular	SARS-CoV-2 spike	AIM Innovation Biotechnology	Clinical trial III NCT05428592	^239^
Novavax	COVID-19, LNP	Intramuscular	SARS-CoV-2 spike	Moderna	Clinical trial III NCT05658523	^240^
LVRNA009	COVID-19, LNP	Intramuscular	SARS-CoV-2 spike	AIM Innovation Biotechnology	Clinical trial III NCT05682638	^239^
BNT162b2	COVID-19, LNP	Intramuscular	SARS-CoV-2 spike	Pfizer	Clinical trial III NCT05749926	^238^
LVRNA021	COVID-19, LNP	Intramuscular	SARS-CoV-2 spike	AIM Vaccine Co., Ltd.	Clinical trial III NCT05812014	^240^
mRNA-1283.222	COVID-19, LNP	Intramuscular	SARS-CoV-2 spike	Moderna	Clinical trial III NCT05815498	^240^
mRNA-4157	Cancer stage III/IV melanoma, LNP	Intramuscular	Neoantigen	Moderna and Merck	Clinical trial IIb NCT03897881	^126^
BNT112	mCRPC/LPCLNP	Intravenous	4 cancer antigens	BioNTech RNA Pharmaceuticals	Clinical trial II NCT04382898	^95^
BNT141	GC/PC/OC/BTC, LNP Multiple solid tumors	Intravenous Intramuscular	CLDN18.2	BioNtech	Clinical trial II NCT04683939 Clinical trial I/IIa	^241^
mRNA-1893	Flavivirus, LNP	Intramuscular	Zika	Moderna	Clinical trial II NCT04917861	^242^
mRNA-1647	CMV, LNP	Intramuscular	gB	Moderna	Clinical trial II NCT05085366	^243^
mRNA-1189	EBV, LNP	Intramuscular	EBV gB (gB/gH/gL/ gp42/gB350)	Moderna	Clinical trial I NCT05164094	^244^
mRNA-1345	RSV-LRTD, LNP	Intramuscular	preF glycoprotein	Moderna	Clinical trial II NCT05085366	^245^
mRNA-1574	HIV, LNP	Intramuscular	BG505 MD39.3/BG505 MD39.3 gp151/BG505 MD39.3 gp151 CD4KO	National Institute of Allergy and Infectious Diseases (NIAID)	Clinical trial I NCT05217641	^246^
mRNA-1215	Nipah virus, LNP	Intramuscular	pre-F/G	National Institute of Allergy and Infectious Diseases (NIAID)	Clinical trial I NCT05398796	^246^
mRNA-1644	AIDS, LNP	Intraperitoneal	eOD-GT8 60mer	International AIDS Vaccine Initiative	Clinical trial I NCT05414786	^247^
mRNA-1010	Candidate variations Influenza	Intramuscular	HA	Moderna	Clinical trial II NCT05868382	^248^

**Fig. 4:**
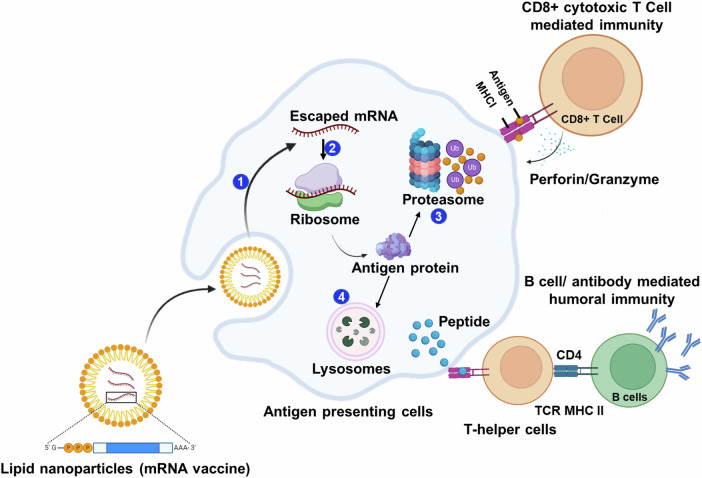
Mechanism of mRNA vaccine. (1) The mRNA vaccine is delivered into antigen-presenting cells by lipid nanoparticle (LNP). (2)The mRNA encoding the disease-targeted spike protein is released into the cytoplasm and translated into the antigen protein by the ribosome. (3) Some antigen proteins are degraded into small peptides by the proteasome and presented to the surface of CD8+ T cells by major histocompatibility complex I (MHCI). The CD8+ cytotoxic T-cell-mediated immunity kills infected cells by secreting perforin or granzyme. (4) Other antigen proteins are degraded in the lysosome and displayed on the surface of T helper cells by MHC II. The B-cell/antibody-mediated humoral immunity uses antibodies to neutralize pathogens(Created with BioRender.com).

Early attempts to use IVT mRNA for medicinal purposes helped generate highly effective SARS-CoV-2 mRNA vaccines quickly. Early attempts to use IVT mRNA for medicinal purposes helped to generate highly effective SARS-CoV-2 mRNA vaccines^81^. Before the end of 2019, several preclinical and clinical investigations had demonstrated the potential of mRNA vaccines to protect against a range of diseases, including the rabies virus, influenza A virus, respiratory syncytial virus (RSV), and Zika virus. But it was expected to take another 5–6 years before an mRNA vaccine was approved for use in clinical settings^81^. This process was accelerated by the COVID-19 pandemic, which led to the approval of mRNA vaccines created by Moderna and BioNTech/Pfizer in just 10 months. These vaccines transmit nucleoside-modified mRNA encoding the viral spike glycoprotein. They are made with ionizable LNPs. Studies with patients showed success rates of over 90%. There were brief local and systemic reactions, but no serious safety issues^83,84^. However, continued assessment is crucial to address potential long-term effects. Early attempts to utilize IVT mRNA for therapeutic purposes paved the way for the rapid development of highly efficient mRNA vaccines against SARS-CoV-2^81^. The mechanism of mRNA vaccines is represented in Fig. 4.

mRNA vaccines are typically administered via a single injection into the skin, muscle, or subcutaneous space, where they induce an immune response by producing antigens through translation in immune or non-immune cells. Unlike plasmid DNA and viral DNA vectors, IVT mRNA does not require entry into the nucleus to be effective. The immune system has high sensitivity, enabling strong immune responses even with low antigen levels and eliminating the necessity for high and sustained IVT mRNA expression. The mRNA platform offers various advantages for pandemic vaccine production, including rapid development and cost-effective, scalable production, allowing quick responses to emerging pandemic viruses. Additionally, it permits flexible antigen design and the delivery of multiple antigens in a single formulation, facilitating the creation of “universal” vaccines that provide broad protection against diverse viral strains. Despite the need for further development in regulatory and approval processes for mRNA vaccines, more mRNA vaccines for infectious diseases are expected to emerge in the near future.

Another exciting use of mRNA vaccines is in personalized cancer therapy, where current clinical trials show promise in triggering immune responses against cancer-specific neoantigens, with initial results suggesting potential clinical advantages^85–87^. These vaccines are customized for each patient using RNA sequencing data from their tumor tissue, enabling the immune system to specifically recognize and attack cancer cells^88^. Additionally, mRNA vaccines also hold promise for treating autoimmune diseases by selectively dampening harmful autoimmune reactions without affecting normal immune activity. This is accomplished by systemically delivering mRNA that encodes specific autoantigens associated with the disease^89^. However, challenges persist in utilizing therapeutic mRNA to express proteins that are absent or dysfunctional in the body, including the necessity for sustained protein expression, difficulties in delivering mRNA to solid organs, and the potential for immune activation and reduced therapeutic effectiveness with repeated dosing. Despite these challenges, some clinical studies, such as the use of VEGF mRNA for cardiac regeneration, have shown promising safety and efficacy outcomes^90^.

The emergency use and subsequent approvals of mRNA vaccines BNT162b2 and mRNA-1273 provided the strongest clinical validation of IVT mRNA platforms, demonstrating high efficacy and acceptable short-term safety in large randomized trials and enabling rapid scale-up of GMP manufacturing. Beyond infectious disease, early-phase clinical work now explores mRNA for regenerative and oncologic applications: intramyocardial VEGF-A mRNA (AZD8601) was safely administered during cardiac surgery and showed encouraging biological signals in Phase I/II work, supporting tissue-targeted protein replacement strategies. Personalized neoantigen mRNA vaccines indicate the feasibility of individualized mRNA cancer vaccines that induce tumor-specific T-cell responses and can be integrated with standard therapies in early trials. These examples collectively show that mRNA therapeutics are moving steadily from prophylactic vaccines toward therapeutics that demand precise delivery, patient-specific manufacture, and careful immune-monitoring.

### CRISPR-Cas-mediated genome editing

**Fig. 5:**
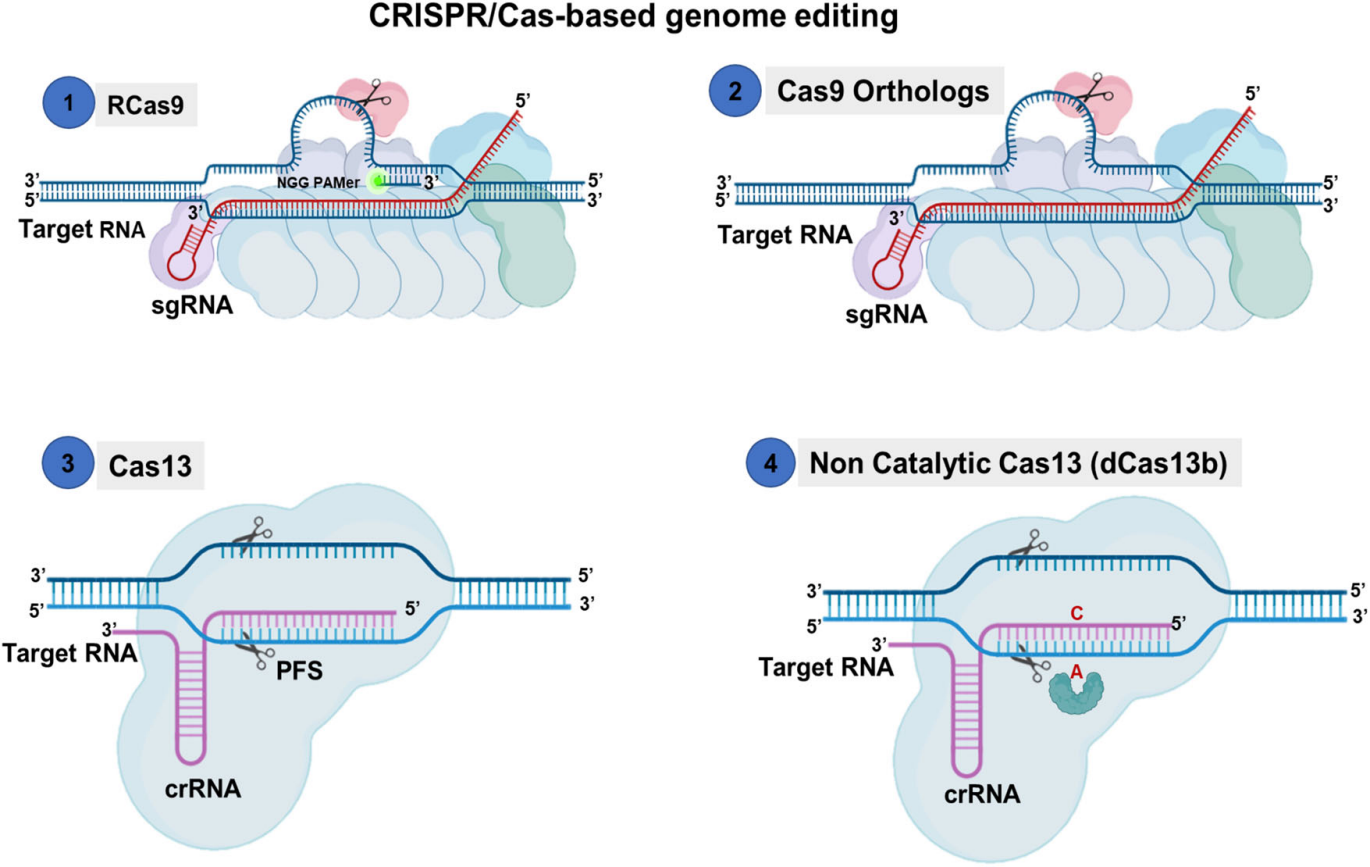
CRISPR/Cas-based gene editing mechanism. The CRISPR/Cas RNA editing system involves two types of Cas nucleases: Cas9 and Cas13. A guide RNA (gRNA) is associated with Cas9 to cut ssRNA either with (1) or without (2) the need for a protospacer-adjacent motif (PAM). (3) Cas13 is directed by a single CRISPR RNA (crRNA) to target specific RNA containing a protospacer flanking sequence (PFS). (4) Beyond knocking down target RNA, a catalytically inactive Cas13b (dCas9b) enables A-to-I editing with the help of ADAR (Created with BioRender.com).

In their natural environment, bacteria and archaea utilize RNA-guided endonucleases from various CRISPR-associated protein (Cas) systems to recognize and cleave foreign nucleic acids as part of their adaptive immune system^91^. These systems maintain a memory of previously encountered pathogens by storing nucleic acid sequences captured during past infections, which are then employed as “spacer sequences” to guide CRISPR–Cas proteins in targeting and destroying the DNA or RNA of future pathogens (as represented in Fig. 5). This mechanism allows CRISPR–Cas systems to be easily reprogrammed to target diverse DNA or RNA sequences by utilizing different spacer sequences within a guide RNA molecule, provided that the corresponding target DNA “protospacer” sequence is located adjacent to a suitable Protospacer-Adjacent Motif (PAM)^92,93^. A PAM is necessary to safeguard the genomic DNA containing guide RNA sequences from destruction by CRISPR–Cas systems, as these sequences include targeted spacers but lack adjacent PAM sequences^91^.

The Cas systems, originally derived from prokaryotes, have found widespread use in mammalian cells and organisms for precise genome editing, leading to the permanent knockout or alteration of a targeted gene. This system operates through a designed guide RNA (gRNA) and an RNA-guided Cas nuclease, where the gRNA forms a complex with Cas, recognizing a specific sequence along with a protospacer adjacent motif (PAM) element. Subsequently, the Cas nuclease cleaves either double-stranded DNA (dsDNA) or single-stranded RNA (ssRNA) at the designated site for efficient genome editing^94^. Initial successes have spurred the development of novel methods for nucleic acid targeting and manipulation, including those derived from Cas9 and Cas13 orthologs^95^. The Cas9 system, for example, can target both double-stranded DNA (dsDNA) and single-stranded RNA (ssRNA), requiring a corresponding guide RNA (gRNA) and a complementary PAM-containing oligonucleotide (PAMmer) specific to Streptococcus pyogenes RCas9^96^.

Moreover, Cas9 orthologs from organisms like *Campylobacter jejuni* and *Staphylococcus aureus* possess the ability to cleave ssRNA without the need for a PAM^97^. Additionally, Cas13-based systems specifically target RNA, using a CRISPR RNA (crRNA) to direct the cleavage process. Different Cas13 variants, including Cas13a, Cas13b, and Cas13d, have been shown to effectively disrupt and silence target RNAs in mammalian cells in vitro, with CasRx (RfxCas13d) displaying especially strong RNA knockdown efficiency in HEK293T cells^98^. Additionally, Cas13b, in a catalytically inactive form, can promote A-to-I base conversion by fusing with the ADAR2 deaminase domain (ADAR2DD), whereas Cas13d provides a unique PFS-independent Cas variation^99^. The list of CRISPR/Cas9-based therapies that have received FDA approval or are undergoing clinical evaluation is provided in Table 4.

**Table 4:** CRISPR/Cas9 with either FDA approval or in clinical trials

Drug/alternative name	Target organ/disease	Dose/route	Key observation	Organization	Updated status	References
Casgevy	Sickle cell disease and β-thalassemia	Intravenous	---	Vertex Pharmaceuticals and CRISPR Therapeutics	First gene therapies to treat patients with sickle cell disease	^249^
CARBON	Relapsed/refractory B-cell malignancies (TRAC and B2M Allogeneic T cells)	Intravenous infusion following lymphodepleting chemotherapy.	CTX110: CD19-directed allogeneic T cells genetically modified ex vivo using CRISPR-Cas9 gene editing components	CRISPR Therapeutics AG	Clinical trial I NCT04035434	^250^
NCT05643742	Relapsed/refractory B-cell malignancies Regnase-1, TGFβR2, TRAC, B2M	Intravenous infusion following lymphodepleting chemotherapy.	CTX110+disruption of Regnase-1 and TGFβR2 via CRISPR/Cas9	CRISPR Therapeutics AG	Clinical trial 1/2 NCT05643742	^251^
Voretigene neparvovec/Luxterna	Retinal dystrophy	Subretinal injection	--	Spark Therapeutics, Inc.	2017 FDA approved	^252^

#### Limitations

Technical constraints and advancements in CRISPR technologies have raised concerns regarding immunogenic toxicity. A recent study revealed that many human subjects possessed pre-existing antibodies against Cas9, a commonly used bacterial nuclease. Specifically, more than 50% of the subjects exhibited immunity against two extensively studied nucleases, SaCas9 and SpCas9, which are prevalent in human blood, eliciting an immunogenic response^100^. These findings underscore the importance of conducting thorough investigations, particularly in vivo gene therapy applications. Moreover, the guide RNA (gRNA) can trigger an innate immune response in human cells due to a phosphate group at the 5’ terminal^101^. Although CRISPR has been extensively utilized in clinical trials to modify somatic cells ex vivo, aiming to mitigate risks before transferring them for in vivo gene therapy, ethical challenges persist in germ-line gene editing studies for therapeutic purposes. Consequently, ongoing and upcoming clinical trials on somatic CRISPR therapy require careful long-term evaluation to comprehensively assess system efficacy and safety.

CRISPR technologies have rapidly advanced from preclinical promise to clinical reality. Ex vivo editing programs (for example, CTX001/exa-cel, now marketed as Casgevy) that edit autologous hematopoietic stem cells have shown transformative outcomes in sickle cell disease and transfusion-dependent β-thalassemia, demonstrating durable clinical benefit but also highlighting logistical and manufacturing complexities inherent to cell-based gene editing. In vivo programs (e.g., EDIT-101 for LCA10) have delivered CRISPR payloads directly to target tissues and shown early signs of biological activity with acceptable safety profiles, illustrating the feasibility of local in vivo editing. These clinical case studies reveal common translational constraints manufacturing scale, delivery specificity, pre-existing anti-Cas immunity, and long-term follow up that must be addressed as RNA-guided editing moves toward broader clinical deployment.

A notable limitation of genome-editing approaches lies in the inherent instability of mRNA molecules encoding large CRISPR/Cas systems. The substantial size of CRISPR-associated nuclease transcripts, such as SpCas9, significantly influences their susceptibility to degradation and poses challenges for efficient delivery and translation in target cells^102^. Larger mRNA constructs are more prone to enzymatic breakdown, demonstrate reduced encapsulation efficiency within lipid or polymeric nanocarriers, and often exhibit limited in vivo stability^103^. Moreover, simultaneous delivery of nuclease mRNA with guide RNA or repair templates further complicates formulation design and dosing strategies. To address these challenges, recent studies have explored the development of compact Cas variants (e.g., SaCas9, Cpf1), split-Cas systems, and optimized nanocarrier formulations that enhance mRNA protection and intracellular release^104^. Despite these advances, ensuring mRNA stability and delivery efficiency for large CRISPR constructs continues to represent a major barrier to the successful clinical translation of mRNA-based genome editing therapies^105^.

## Delivery strategies for RNA-based therapeutics

Overcoming biological barriers is crucial for efficiently delivering RNA-based therapeutics, which face numerous challenges in reaching their intended targets. Thus, here is an elaboration on strategies aimed at addressing these challenges and fostering innovations in RNA delivery.

### Enhanced stability

RNA molecules are inherently unstable and prone to degradation by nucleases present in bodily fluids^106^. To mitigate this limitation, extensive research has focused on chemical and structural optimization of RNA molecules. Incorporation of nucleoside modifications such as 2′-O-methyl, 2′-fluoro, pseudouridine, and N1-methyl-pseudouridine has been shown to markedly enhance resistance to enzymatic hydrolysis while simultaneously improving translational efficiency^107^. Similarly, optimizing RNA secondary structure and codon usage using computational tools such as LinearDesign has proven effective in prolonging mRNA half-life and increasing protein expression without introducing new immunogenic motifs^108^. Beyond intrinsic modifications, encapsulation of RNA within protective carriers such as LNPs, lipid-polymer hybrids, or biodegradable polymers provides a steric shield against nuclease attack. These carriers maintain molecular integrity during systemic circulation and facilitate controlled release at the target site^109^. Surface modification with hydrophilic polymers, most notably polyethylene glycol (PEG), further prevents opsonization and clearance by the mononuclear phagocyte system, thereby extending circulation time and bioavailability^110^.

### Improved cellular uptake

Efficient intracellular delivery is another critical determinant of RNA therapeutic efficacy. Following systemic administration, nanoparticles interact with plasma proteins and are internalized by cells primarily through endocytic pathways such as clathrin-mediated or caveolae-mediated endocytosis^111^. However, once inside endosomes, RNA must escape into the cytoplasm before lysosomal degradation. Ionizable lipids that become positively charged in acidic environments, together with helper lipids like DOPE, enable endosomal membrane destabilization through the proton-sponge or fusion mechanism, leading to efficient cytosolic release^112^. Additionally, cell-penetrating peptides (CPPs) such as TAT, penetratin, and arginine-rich sequences have been explored for enhancing uptake, either through direct translocation or receptor-mediated processes^113^. Recent studies also highlight the importance of tuning nanoparticle surface charge and pKa: slightly cationic or near-neutral surfaces minimize non-specific protein binding while promoting efficient cellular entry^114^.

### Targeted delivery

Targeted RNA delivery remains a major challenge yet holds the key to maximizing efficacy and minimizing systemic toxicity. Innovations in targeting ligands and receptor-mediated delivery systems enable precise localization of RNA therapeutics to desired sites within the body. By selectively targeting diseased cells or tissues, these delivery strategies enhance therapeutic efficacy while reducing systemic toxicity^115^. Passive accumulation through the enhanced-permeability-and-retention (EPR) effect allows nanoparticles within 50–150 nm to concentrate in tumors and inflamed tissues, although this mechanism shows variability in humans^116^. Active targeting employs receptor-specific ligands such as N-acetylgalactosamine (GalNAc) for hepatocytes, folate for cancer cells, and antibodies or aptamers for precision delivery to immune or endothelial cells. For instance, GalNAc-conjugated siRNA therapeutics, including givosiran and inclisiran, have achieved clinically validated liver-specific gene silencing. Furthermore, emerging biomimetic approaches exploit the adsorption of endogenous proteins such as Apolipoprotein E (ApoE) onto LNP surfaces to promote receptor-mediated hepatic uptake. In immunotherapy applications, targeted mRNA delivery to dendritic cells via receptor-specific ligands has been shown to amplify antigen presentation and improve vaccine potency^117^.

### Immune response

The activation of innate immune responses by RNA molecules poses a hurdle to their successful delivery. Strategies to modulate immune responses and minimize inflammatory reactions include the design of RNA molecules with reduced immunogenicity, as well as the incorporation of immunomodulatory agents into delivery systems. These innovations help circumvent immune activation, thereby improving the safety and tolerability of RNA-based therapies. The innate immune system readily recognizes exogenous RNA through pattern-recognition receptors such as TLR3, TLR7/8, and RIG-I. Activation of these pathways can lead to cytokine release, inflammation, and diminished therapeutic efficacy^118^. To mitigate such immune responses, modified nucleotides like pseudouridine and 5-methylcytidine are widely used to suppress TLR activation while maintaining translational competence. Encapsulation within biocompatible carriers such as LNPs and polymeric vesicles also shields RNA from immune recognition. In addition, formulations incorporating immunomodulatory agents such as dexamethasone or anti-inflammatory lipids can further reduce local inflammation and improve tolerability. Optimization of lipid composition is required to minimize complement activation or pseudoallergic reactions occasionally observed with cationic lipids^119^.

### Biocompatibility and safety

Ensuring the biocompatibility and safety of RNA delivery systems is paramount for clinical translation. Innovations in the design of delivery vehicles focus on using biocompatible materials and minimizing cytotoxicity. Additionally, advancements in formulation techniques aim to optimize the pharmacokinetics and biodistribution of RNA therapeutics, maximizing their therapeutic potential while reducing adverse effects^120^. Ionizable lipids that degrade into neutral metabolites, biodegradable polymers such as poly(β-amino esters), and zwitterionic coatings are now preferred to avoid chronic tissue accumulation^121^. Biodegradability also mitigates hepatosplenic buildup observed after repeated dosing of conventional. Furthermore, rational design of nanoparticle pharmacokinetics, balancing circulation half-life, tissue penetration, and clearance, remains essential for achieving therapeutic efficacy without off-target toxicity. Current clinical data from mRNA vaccines and siRNA therapeutics indicate that well-optimized delivery systems can achieve high potency with acceptable safety profiles, underscoring the promise of continued material innovation in RNA medicine^122^.

### Combination therapies

The clinical success of RNA therapeutics underscores a key translational principle: the mode of delivery largely determines the therapeutic indication. Among current platforms, ionizable LNP have been pivotal in enabling in vivo delivery of mRNA, as demonstrated by the rapid development of the COVID-19 vaccines BNT162b2 and mRNA-1273^83,84^. These LNP efficiently encapsulate and transport mRNA into antigen-presenting cells following intramuscular injection, driving strong protein expression and robust immune responses. In contrast, N-acetylgalactosamine (GalNAc) conjugation has proven ideal for hepatocyte-targeted siRNA drugs, such as givosiran and inclisiran, where receptor-mediated uptake via the asialoglycoprotein receptor allows potent and sustained gene silencing after infrequent subcutaneous dosing^123,124^.

Additionally, local delivery approaches, including intracoronary or intramyocardial administration of *VEGF-A mRNA* (e.g., AZD8601), have demonstrated safety and biological activity in early-phase cardiac-repair trials^125^. Collectively, these examples show that delivery modality rather than RNA sequence alone governs biodistribution, dosing frequency, safety profile, and ultimately the clinical applicability of each RNA platform.

Synergistic approaches that combine RNA-based therapeutics with orthogonal modalities are rapidly maturing and provide practical routes to surmount biological barriers and improve outcomes. A leading clinical example is the combination of a personalized neoantigen mRNA vaccine with PD-1 blockade: the mRNA-4157 (V940) vaccine administered with pembrolizumab produced clinically meaningful improvements in recurrence-free and distant-metastasis-free survival in resected high-risk melanoma (KEYNOTE-942/mRNA-4157-P201), illustrating how RNA vaccines can be potentiated by immune-checkpoint inhibition to augment antitumour immunity^126^. In oncology and beyond, RNA agents are being deployed alongside small molecules that modulate target biology or the tissue microenvironment, for example, pairing RNA therapeutics that reduce pathogenic transcript abundance with small-molecule pathway inhibitors to create complementary on-target suppression and pathway blockade (for reviews of RNA-targeting small molecules and RNA small-molecule strategies, see recent reviews)^127,128^.

Gene editing modalities also provide synergistic opportunities: ex vivo CRISPR editing combined with RNA delivery (e.g., gRNA/mRNA delivery to engineered cells) enables durable cellular therapies (for instance, CRISPR-edited autologous HSC and CAR-T programs), and in vivo combinations of editing and transient RNA expression may allow correction plus temporary supportive protein expression^129^. Finally, advances in delivery chemistry, such as LNP optimization, biodegradable ionizable lipids and cell-targeting ligands, can be paired with immune-modulatory agents to reduce toxicity while enhancing uptake and endosomal escape, thereby improving the therapeutic index of combination regimens^130,131^. Together, these examples demonstrate that rationally designed combination strategies informed by mechanism, delivery and patient biology are a promising pathway to overcome delivery, immunogenicity and efficacy barriers for RNA therapeutics.^132^

## Various delivery platforms for RNA therapy

The development of reliable carriers that can shield RNA molecules from harsh physiological conditions is critical, given their inherent instability, negative charge, and susceptibility to enzymatic degradation. Advances in nanotechnology and materials science have provided innovative solutions for overcoming these challenges by improving protection, circulation time, and cellular uptake. A flexible and efficient delivery platform can be achieved by tailoring both the physical properties of nanoparticles (such as size, shape, and chemical composition) and their biological characteristics (such as surface ligands for targeted delivery). Various RNA delivery systems have been developed using both viral and non-viral vectors, including LNPs, polymeric carriers, and dendrimers^133,134^. While viral vectors offer high transfection efficiency, they are costly, have limited packaging capacity, and raise biosafety concerns. In contrast, non-viral vectors are safer, more economical, and capable of accommodating a broader range of RNA cargos. The following section briefly discusses the major types of RNAi delivery platforms, their recent advancements, and the key challenges that must be addressed for successful clinical translation^135^.

### Viral gene therapies

Viral gene therapies have demonstrated promising clinical outcomes, as evidenced by successful clinical trials^136^. The success of these methods can be limited by various factors, such as pre-existing immunity, virus-triggered immunogenicity, potential risks of unintended genomic integration, restrictions on payload size, difficulties with repeated dosing, challenges in large-scale production, and the high costs of vector manufacturing^137,138^. Despite ongoing efforts to address some of these limitations and enhance the safety and efficacy of viral gene therapies, these challenges have spurred the exploration of alternative drug delivery platforms^139^.

Researchers are actively seeking novel drug delivery vehicles that can overcome the drawbacks associated with viral vectors. These alternative delivery systems offer the potential to circumvent immune responses, minimize off-target effects, accommodate larger therapeutic payloads, facilitate repeated dosing regimens, simplify manufacturing processes, and reduce overall treatment costs^140^. By harnessing innovative technologies and engineering approaches, scientists aim to develop next-generation delivery platforms that can efficiently and safely deliver therapeutic genes to target cells and tissues, ultimately advancing the field of gene therapy and expanding its clinical utility^141^.

### Lipid nanoparticles (LNPs)

LNPs are the most frequently utilized carriers for administering oligonucleotide medications, serving as comprehensive delivery systems. These LNPs comprise ionizable cationic lipids, cholesterol, phospholipids, and PEG-lipids. The core constituents, ionizable cationic lipids, form “lipoplexes” through electrostatic interaction with negatively charged nucleic acids, facilitating nucleic acid transfections in vitro, such as Lipofectamine™ RNAiMAX transfection reagents^142^. Helper lipids, phospholipids, and cholesterol are essential components that significantly improve the stability of formulations and enhance delivery effectiveness^143^. Furthermore, PEG-lipids regulate particle size, prevent aggregation, and prolong in vivo circulation durations, contributing to their versatility^144^.

The ionization behavior and surface charge of LNPs play a crucial role in mediating siRNA delivery, which is influenced by factors such as the acid dissociation constant (pKa). Positively charged LNPs coat anionic RNAs, facilitating their cell entry via receptor-mediated endocytosis and preventing nuclease degradation. Upon entering cells, LNPs undergo a decrease in pH, resulting in an increase in nanoparticle charge due to protonation and subsequent buffering capacity within endosomes. This buffering capacity contributes to endosome swelling and breaking, facilitating siRNA release into the cytosol, a critical step for its therapeutic action through RNA interference^145^. LNPs enable the delivery of various RNA entities, including mRNA and CRISPR–Cas components, to specific cells and tissues using advanced technologies like selective organ targeting (SORT), enabling tailored gene therapies for hepatic and rare diseases^146^. Moreover, optimizing LNPs’ pKa enhances RNA delivery efficiency, while branched-tail LNPs demonstrate the capability to co-deliver multiple mRNAs without inducing immunogenicity or toxicity^147^. Overall, LNP-based gene therapies are promising for treating hepatic diseases and other rare disorders, showcasing their potential for widespread application in RNA therapeutics.

LNPs represent a vital class of drug delivery systems, with FDA-approved variants demonstrating efficacy in liver siRNA delivery and mRNA vaccine administration^148,149^. The distinct structures of lipids within LNPs influence their interactions with cells, and researchers have extensively investigated lipid-based delivery systems to optimize the delivery of nucleic acids. Through various synthetic methods, scientists have developed diverse libraries of lipid delivery systems, with a particular focus on enhancing siRNA delivery to hepatocytes in mice. These efforts, coupled with a more targeted approach to lipid design, have significantly reduced the required dose for effective in vivo hepatocyte gene silencing in preclinical models^150,151^.

**Fig. 6:**
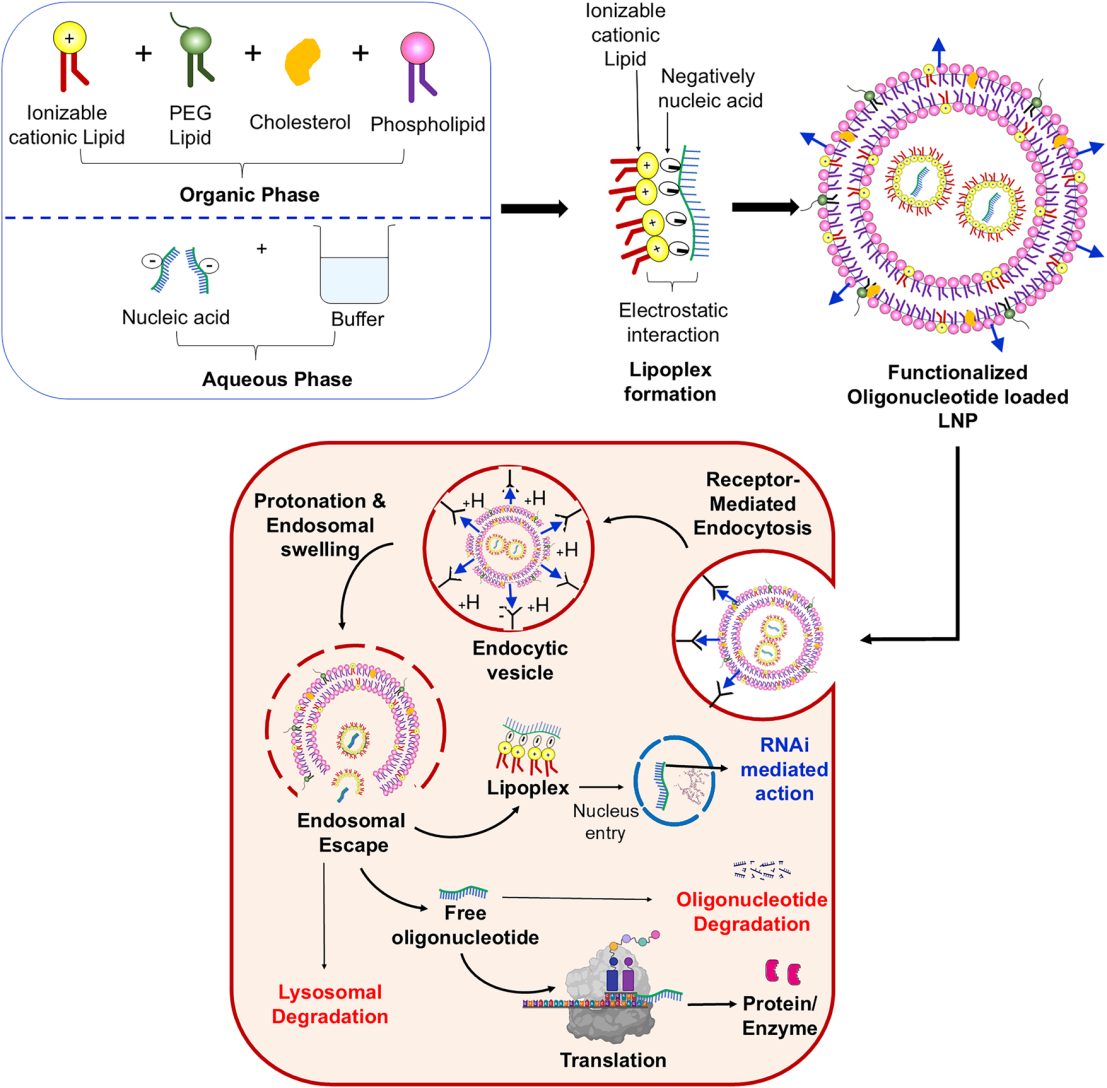
Lipid nanoparticle–mediated intracellular delivery of oligonucleotides. Schematic illustration of oligonucleotide-loaded lipid nanoparticles showing cellular uptake via endocytosis, endosomal escape facilitated by ionizable lipids, and subsequent cytoplasmic release for RNA interference–based gene silencingc.

In summary, LNPs represent a versatile and promising platform for delivering RNA therapeutics. Key lipids, such as C12-200, cKK-E12, DLin-KC2-DMA, and DLin-MC3-DMA, have demonstrated efficacy in providing siRNA in non-human primates, with DLin-MC3-DMA notably utilized in the patisiran treatment for hATTR^130^. LNPs have also successfully delivered mRNA to the liver in various preclinical and clinical settings, showcasing their potential for widespread application. Recent advancements, including LNPs delivering base-editing Cas9 and sgRNA targeting PCSK9, have shown sustained gene silencing effects in non-human primates, with potential therapeutic benefits for cardiovascular diseases^152^. Figure 6 provides a detailed representation of LNP-mediated delivery of oligonucleotides and their intracellular fate.

### Polymers and polymer-based nanoparticles

Numerous RNA delivery methods that do not involve viruses also rely on polymers and polymeric nanoparticles. Chemists can modify polymer properties like charge, degradability, and molecular weight, which influence how effectively RNA is delivered into cells^153^. A commonly employed polymer is poly (lactic-co-glycolic acid) (PLGA). While the FDA has authorized PLGA drug delivery systems for administering small-molecule drugs, they have not yet approved them for nucleic acid delivery^154^. PLGA lacks the positive charge to bind to the negatively charged RNA phosphodiester backbone at a neutral pH. Therefore, scientists have modified PLGA by incorporating cationic chemical groups like chitosan to enable the delivery of siRNA in mice^155^.

PLGA lacks the positive charge necessary to form complexes with the negatively charged RNA phosphodiester backbone at a neutral pH. Researchers have introduced cationic chemical groups like chitosan to address this limitation and utilize PLGA for RNA delivery, enabling effective siRNA delivery in mice^154^. Polymers containing amine groups capable of becoming cationic, such as polyethylenimine (PEI) and poly(l-lysine) (PLL), can interact electrostatically with RNA, facilitating its cellular uptake^156^. However, unmodified PEI and PLL may not always be well tolerated, with PEI transfection efficiency and toxicity increasing with molecular weight^157^. Consequently, chemical modifications have been applied to enhance the in vivo performance and tolerability of PEI and PLL. For instance, nanoparticles incorporating PEG-grafted PEI have been employed to transport mRNA to immune cells in the lungs. At the same time, cyclodextrin-PEI conjugates have been utilized for in vivo mRNA vaccine delivery^158^.

Similarly, surface modification of iron oxide nanoparticles with PLL has enabled gene delivery to the central nervous system in mice^159^. Comparing poly(ethylene glycol) and poly(lactic lactate) to another class of cationic polymers, poly(beta-amino esters) (PBAEs), which are created by joining amine monomers to diacrylates, provides better biodegradation and less cytotoxicity^160^. These polymers were created to improve the delivery of RNA and DNA. They feature biodegradable ester linkages and cationic amines^161^. Initially, a variety of chemically different PBAEs were produced using Michael addition chemistry, and their effectiveness in delivering DNA and RNA in cell culture environments was evaluated^162,163^.

These PBAE ‘libraries’ facilitated investigations into how the chemical structure of PBAEs impacts drug delivery, leading to the establishment of design principles for subsequent PBAE development^164^. Initial guidelines suggested that effective polymers tended to be predominantly hydrophobic, containing mono-alcohol or di-alcohol side groups, and featuring linear bis(secondary) amines. A subsequent study employing these design criteria revealed that the most successful polymers predominantly comprised amino alcohols and exhibited chemical similarities, differing only by one carbon atom^164^. These studies demonstrated the viability of using high-throughput chemical synthesis and then high-throughput drug delivery evaluations; this approach has been applied to other chemical classes of nanoparticles, such as LNPs^165^. PBAEs have been utilized for delivering DNA vectors to pulmonary cells via nebulization, mRNA intranasally, and siRNA to a human orthotopic glioblastoma tumor model in mice^166,167^. More recently, PBAE-based polymers have been used by researchers to nebulize Cas13a mRNA and guide RNA into the respiratory tracts of mice and hamsters for the treatment of SARS-CoV-2^168^. Additionally, scientists have synthesized lipid-polymer hybrids, observing that the addition of lipids to PBAEs enhances serum stability and delivery efficiency^169^.

### Inorganic nanoparticles

Non-viral inorganic NPs have grown into better miRNA and siRNA delivery techniques owing to their variable size, low cytotoxicity, imaging diagnostics, storage stability, targeting site, cross-biological barriers, and excellent pharmacokinetics. The inorganic NPs typically comprised metal nanoparticles (MNPs), magnetic NPs/Iron oxide NPs (IONs), mesoporous silica NPs (MSN), and calcium phosphate NPs etc^170–172^. Inorganic NPs provide an ideal scaffold for the development of RNA-based delivery vehicles for both in vitro and in vivo applications. They possess a high surface area to volume ratio, enabling optimal siRNA loading through either chemical conjugation or non-covalent encapsulation. A key benefit of these NPs has to do with their surface chemistry, which might be easily altered, allowing for the resolution of issues regarding in vitro and in vivo siRNA delivery. Additionally, the varied physical and optical characteristics of inorganic NPs can be exploited to track siRNA distribution to cells or tissues^173^. The negatively charged nucleic acids need to be bound and encapsulated by the cationic biomaterial transfection agent. Consequently, MNPs, which carry the positive charge, are the assigned cationic biomaterials for safe and effective intracellular delivery of RNA therapeutics^174^.

#### Gold nanoparticles (AuNPs)

The advanced surface chemistry of AuNPs, permitting the covalent or non-covalent siRNA conjugation, as well as their inert, non-toxic, and biocompatible core, is being explored strongly for siRNA delivery. Considering it, AuNPs are frequently researched along with hollow AuNPs or AuNP-siRNA conjugates as a captivating siRNA delivery system among various MNPs, owing to their optical properties^173^. As the most straightforward technique, thiol-gold covalent chemistry has been employed by Nagasaki and coworkers to deliver siRNA via siRNA-AuNP conjugates^175^. Multiple approaches, particularly amino acid-functionalized AuNPs (AA-AuNPs), mixed-monolayer-protected AuNPs (MM-AuNPs), and layer-by-layer-fabricated AuNPs (LbL-AuNPs), have established efficacy in nucleic acid delivery while preserving their safety and stability^176^. PEI-Au/human telomerase reverse transcriptase (hTERT) siRNA and PEI-Au/hTERTsiRNA@ ZGOC samples in B16F10 cells (a mouse melanoma cell line) displayed the anticancer impact^177^. In a rat model of type 1 diabetes, Pluronic F127 gels containing DsiRNA-AuNPs were found to have beneficial influences on diabetic wounds through fostering and improving angiogenesis and wound healing^178^. In a different manner, PEG-PEI polymer-coated AuNPs efficiently delivered siRNA to pancreatic stellate cells, silencing HSP47 and minimizing pancreatic cancer-associated desmoplasia^179^.

#### Mesoporous silica nanoparticles (MSN)

MSN surfaces can be effectively coupled with siRNA thiolates for transport, and coating MSNs with cationic polymers has been found to be productive. A 10 KD PEI-coated MSNPs were discovered by Nel et al. to be capable of transporting siRNA and inhibiting the production of GFP in modified HEPA-1 liver cancer cells without causing cytotoxicity^180^.

Additionally, plenty of MNP-mediated RNA delivery systems, which target other genes, including Bcl2L12, c-Met, EGFR, PLK1, miRNA-21, VEGF, SSATB1, and Galectin-1, have been designed for glioma gene therapy approaches^170^. Hence, the curative value of inorganic NPs for the delivery of siRNA is being proven in a broad spectrum of disease conditions, suggesting a promising therapeutic future.

### Metal-phenolic networks (MPNs)

To address a few insufficiencies in existing RNA delivery platforms, such as existing inflammation due to cationic components of NPs or restricted organ tropism, the polyphenol networks are stabilized by metal ions. In this way, the metal-phenolic networks (MPNs) are another ongoing, negatively charged metal-organic frameworks that evolved when polyphenols and metal ions coordinate under physiological settings. Because of its distinct architecture and chemical structure, MPNs possess several advantages, including ease of manufacture, excellent biocompatibility, acid sensitivity, and possible drug carrier changes for RNA delivery^181^.

In several kinds of cell lines, the modified mRNA-MPN nanoparticles exhibited improved transfection efficiency than conventional Lipofectamine. Similar to LNPs, they facilitate mRNA transport across multiple animal tissues, such as the liver, kidney, lung, heart, and spleen, as well as protein expression and gene editing in the brain. The noncationic nature, favorable biocompatibility, efficacy, and flexibility of mRNA-MPN NPs render them a suitable substitute for conventional mRNA delivery platforms^172^. In order to overcome issues with mRNA distribution by cationic nanoparticle elements, a study presented a noncationic nanoparticle platform. With the help of metal ions, the platform assembles mRNA into a network of poly(ethylene glycol) and polyphenol. Assessing a variety of components and respective compositional ratios yields a library with the ability to achieve stable, biocompatible, and robust mRNA transfection both in vitro and in vivo^172^.

### Dendrimers

Dendrimers are a type of polymer utilized for RNA delivery, characterized by a specific number of branched monomers extending from a central core molecule. Amphiphilic dendrimer vectors share two important features with their chemical hydrophilic counterparts: RNA binding and RNA complex-stabilizing hydrophobicity. It promotes RNA encapsulation inside an established complex, resulting in an equilibrium that influences the efficacy of RNA delivery followed by endocytosis^135^. Dendrimers, an innovative form of cationic polymer, having a positive charge, enable electrostatic interactions with RNA molecules, stabilizing them and inhibiting enzyme degradation in systemic circulation^182^. Functionalised dendrimers facilitate the RNA uptake and minimize off-target effects while enhancing targeted distribution to cells or tissues. Cellular absorption and endosomal release have also been enhanced by their nanoscale size and tunable surface chemistry^183^.

#### Poly(amidoamine) (PAMAM)

The cationic functionality of dendrimers, such as poly(amidoamine) (PAMAM) or poly-L-lysine (PLL), is capable of forming complexes that enhance the delivery of RNA into cells. They have been shown to be critically important in delivering RNA to the CNS, serving as intramuscular vaccines, and transporting siRNA to hepatic endothelial cells^184^. The cellular internalization mechanism describes how dendrimers interact with RNA, resulting in cells taking up the biological and exogenous compounds. A study compared siRNA and PAMAM dendrimer complexes to siRNA and branched polyethylenimine (bPEI) complexes, finding that PAMAM-siRNA complexes showed better cellular uptake. Since dendrimers are internalized by an adsorptive endocytosis pathway, membrane adsorption and an adsorptive endocytosis mechanism are necessary for the effective uptake of PAMAM and PAMAM-siRNA complexes^182^. Bohr et al. investigated the potency of PAMAM dendrimers, which are extensively researched for nucleic acid delivery, in pulmonary distribution through generating dendriplexes with siRNA. These stable complexes confirmed the potent gene silencing as well as substantial cellular uptake in macrophages, signifying that they are capable of more efficient distribution of local RNA therapeutics^182^. Additionally, different kinds of cells readily internalize dendrimers and dendrimer-based nanoformulations via the ATP-powered clathrin-mediated endocytosis pathways. Modifications to dendrimer structure have been implemented to shield nucleic acids from enzymatic degradation and enhance their escape from endosomes^185^. PAMAM dendrimers could be chemically modified to enhance conventional dendrimers with minimized cytotoxicity. Recent research conducted by Joubert et al. showed enhanced mRNA condensation through gene transfection in vitro by effectively producing dendriplexes with mRNA^186^. The appealing features of dendrimers are now increasingly explored for potential carrier for mRNA-based vaccines, along with delivering drugs. Chahal et al. explored the use of a modified PAMAM dendrimer molecule to deliver mRNA replicons against Toxoplasma gondii, H1N1 influenza, and the Ebola virus^187^. Thus, dendrimers seem to have increased therapeutic value in a variety disease through modulating immunological and metabolic pathways as well as regulating gene expression via RNA delivery.

### Cyclodextrins

Cyclodextrins (CDs), natural cyclic oligosaccharides, are being implemented as delivery vehicle for siRNA, igniting new interest in this area. CDs consist of modified starch derivatives composed of d-glucopyranose units linked by α-(1-4) interactions, which provides them with the appearance of a doughnut or stunted cone shape. Commercially available units of polysaccharides with low toxicity are α-, β-, and γ-CDs. Their distinct three-dimensional arrangement enables multiple compounds to create host-guest inclusion complexes. Among them, β-CD is the most widely utilized and extensively investigated compound, with several tests for toxicity that comply with FDA guidelines. The peculiar structure of β-CDenables different chemical inclusions too for efficient delivery of siRNA^188,189^.

CDs are excellent substitutes for the different cationic non-viral vectors utilized for siRNA delivery due to their 50–200 nm size range. Also, they act as adapter molecules, allowing substances like modified adamantanes to be merely “plugged” within the CD cone to provide extra functionality. By minimizing the utilization of modified siRNA, CDs shield siRNA from serum^190^. By functionalising CDs vectors with adamantane-transferrin (AD-Tf) and AD-PEG conjugates, siRNA is able to be administered to animals at therapeutic dosages suitable for humans. Research into CDP-based siRNA delivery systems for the treatment of various diseases is prompted by the fact that CDP-siRNA nanoplexes have an advantage over other siRNA-lipid complexes in that they do not trigger immune stimulation, while siRNA contains an immune stimulatory motif^189,190^. In order to target the essential cell death proteins Bim and PUMA, which become more abundant during sepsis, Brahmamdam et al. have developed a unique CD-based TfR-targeted delivery vehicle for siRNA treatment^191^. The CDs system effectively delivered siRNA to immune effector cells without creating detectable off-target effects, signifying a substantial improvement in the treatment of infectious and other disorders.

### Albumin nanoparticles

Albumin, a soluble protein comprising 585 amino acid residues, sustains biological functioning across pH ranges 4–9. Its three-dimensional structure enables the distribution of drugs with various characteristics, whilst its amino and carboxyl groups serve as binding sites for polymers or ligands. Albumin nanoparticles (NPs) are a promising RNA delivery technology, notably for cancer therapy, since they are non-toxic, biodegradable, and extremely effective, which permits targeted distribution of RNA molecules to cells or tissues^192^. Energy-dependent albumin NPs permeate cells via clathrin- and caveolae-mediated endocytic pathways, ensuring the integrity of encapsulated nucleic acids and preventing their destruction. siRNA, a gene-silencing technique, relies on carriers such as albumin NPs for stability and efficacy. These nanoparticles promote siRNA internalization in tumor cells via transcytosis^193^.

Encapsulating the appropriate nucleic acids or RNA in albumin-based nanoparticles serves as one of the most common approaches to use albumins like bovine serum albumin (BSA) and human serum albumin (HSA) as a delivery system for RNAi in cancer therapy^192,194^. Numerous approaches, involving desolvation, thermal gelation, emulsification, nanospray drying, and self-assembly, may be employed to create these structures. The most often used technique among all of those is desolvation, which utilizes glutaraldehyde as a cross-linker and ethanol as a desolvating agent. As evidenced by the coating of HSA-LNPs-siRNA against GFP in breast cancer cells, albumin is utilized as a coating agent in lipid-based nanocarriers to improve delivery to target areas and minimize proximity to serum proteins^195^.

### Cell-penetrating peptides (CPPs)

Amino acid sequences designated as CPPs have the ability to enter living cells and contribute to the uptake of a variety of payloads, including RNA, plasmids or even proteins. The CPP delivery vectors are designed to carry biologically relevant payloads, such as siRNA, to intracellular areas that are not typically internalized by cells^196^. The RNAi pathway is sometimes triggered by siRNA, a double-stranded RNA that might silence the production of a particular protein. However, siRNAs have a short half-life in the bloodstream and cannot pass through the plasma membrane. To address these issues, covalent conjugation, complex formation, and CPP-functionalized nanocomplexes are the techniques widely developed and applied to deliver siRNA over the plasma membrane using various CPPs^197^.

Nucleic acids are usually anionic and, upon physical mixing, spontaneously tend to bind with cationic CPPs via electrostatic interactions. A straightforward and reliable technique for conjugating CPPs with nucleic acids is the non-covalent approach, which has been mainly used for nucleic acid delivery and has proven therapeutic potential in a number of disease types, including cancer^198^. Miliotou et al. established the covalent attachment of CPP to mRNA using PFVYLI as a neutral hydrophobic peptide, and the system efficiency of system has been evaluated in vitro without cationic delivery components^199^. CPPs would provide a viable option in the future for the development of mRNA therapeutics and vaccines, considering that they have demonstrated notable effectiveness in both mRNA and siRNA delivery.

### Exosomes (extracellular vesicles)

Exosomes are naturally occurring NPs that act as intercellular messengers for the delivery of RNA, offering accurate loading, safeguarding against degradation, as well as tailored delivery for specific tissues. Exosomes, the small extracellular vehicles (EVs) varying in size from 50 to 150 nm, exhibit prospects for therapeutic applications along with the transportation of ASOs. These membrane-bound particles, typically produced by dividing cells, are capable of carrying effector compounds from donor to recipient cells, thereby influencing cellular responses^200^.

In order to comprehend the functional consequences of exosomes-mediated RNA transfer, one must have extensive knowledge of the packaging mechanism and sorting signals in EVs. It makes use of the two primary approaches, namely the exogenous and endogenous packing. EVs are able to be loaded exogenously afterward cell isolation, utilizing the passive incubation or active procedures such as electroporation, sonication, or freeze-thaw cycling, primarily suitable for small-molecule drug carg^201^. Endogenous loading is an approach in which genetic overexpression in parent cells generates desirable EVs, resulting from elevated intracellular RNA concentration and further EV packaging. This approach permits more precise control over cargo composition and has been implemented to load therapeutic proteins, RNAs, and CRISPR-Cas946 complexes into EVs. The sorting mechanisms, including RNA-binding proteins and membrane proteins during biogenesis of exosomes, exhibit an impact on the selective packing of miRNAs into exosomes^202^.

### RNA-aptamer conjugate system

The nucleic acid oligonucleotides having structural conformations, known as aptamers, have been used in cell targeting to improve delivery to the specific site of action and offer excellent detection capabilities for molecules on the cell surface^203^. RNA-aptamer conjugate systems are being utilized for targeting the delivery of RNA therapies, such as siRNA or modified RNA, either by direct conjugation with them or by incorporating them into NPs. These conjugates have a variety of applications, such as anticancer therapy, gene silencing, along with targeted drug delivery^204^.

The RNA-aptamer conjugate system comprises primarily the aptamers and siRNAs have been used in recent studies to improve RNAi potency, decrease undesirable side effects, and accomplish tailored delivery of siRNA. As nanovesicle-encapsulated formulations build up in the liver, aptamer-siRNA chimaeras (AsiCs) exhibited cell-specific expression suppression for target genes, possibly facilitating extrahepatic delivery of therapeutic siRNA cargo. Through co-transcription of aptamer and siRNA, design strategies seek to further enhance the silencing ability of AsiCs, with the possibility of extending to non-covalent aptamer-siRNA chimerization^205^. Aptamer-shRNA chimaeras consistently demonstrate therapeutic efficacy in a variety of ailments. On the other hand, aptamer-miRNA chimaeras are showing therapeutic efficacy in non-small cell lung cancer and advanced ovarian cancer models^203^.

### Hydrogel for RNAi therapeutics

Hydrogels, a class of soft, water-swollen materials with sustainability, tunable characteristics, and injectability, have recently gained acceptance in RNA therapy because of their ability to modulate the therapeutic release, thereby lowering systemic toxicity and enhancing in vivo efficacy. Hydrogels augment RNA stability, minimize therapeutic reduction, mitigate the off-target toxicities, and fewer dose requirements making them a fascinating approach for RNA-based therapeutic delivery, complementing clinical-stage platforms^206^. Hydrogels for RNA delivery are designed for controlled RNA release either by active or passive mechanisms; continued RNA release or stimuli-responsive RNA release either by pH, enzyme or photo radiation^207^.

Hydrogels are employed in a variety of biological programs, when delivering the RNA therapeutics at diseased site and protect against immune reactions. They can be tailored to maximize efficiency based on pathological conditions of disease, with a special focus on cancer therapy, bone regeneration, heart repair and wound healing. Nano-vectors are retained surrounding the tumor in hydrogel-mediated RNA therapies for cancer therapy, promoting absorption by certain cancer cell lines. This is especially significant for brain tumors, as they are capable of crossing biological barriers such as the BBB. Additionally, hydrogels exhibit higher absorption with enhanced bioavailability and tumor accumulation^206,208^. Ding et al. developed an innovative RNA-triple-helix hydrogel for the management of triple-negative breast cancer. The hydrogel, consisting of CXCR4 RNA-triple-helix and siRNA duplexes, effectively destroyed tumors due to the synergistic action of miRNA-205 and oncomiR-221^209^.

Despite major advances in RNA-based therapeutics, several limitations continue to constrain their precision and clinical translation. Among these, hybridization-dependent off-target effects remain one of the most critical challenges. Even with rational design and chemical stabilization, small RNAs can bind to unintended transcripts with partial complementarity, leading to unwanted gene silencing or activation. The molecular mechanisms underlying these interactions are not yet fully understood and may vary depending on cellular context, RNA abundance, and competing endogenous RNA (ceRNA) networks. Consequently, effects that remain undetectable under steady-state conditions may become amplified under disease-induced stress or altered gene expression^210^.

Current computational prediction tools assist in identifying potential off-targets but have inherent limitations. Most rely on digital datasets that do not fully capture biological complexity, post-transcriptional regulation, or the influence of chemical modifications on binding affinity. Thus, while in silico algorithms such as RNAhybrid, miRanda, and RNA22 have improved specificity, experimental validation remains essential to confirm target selectivity and therapeutic efficacy. The accuracy of these tools is also limited by the quality and quantity of available miRNA–mRNA interaction data and by algorithmic biases in AI-based predictive models^211^.

These molecular and computational constraints not only affect RNA design precision but also explain many of the translational discrepancies observed between preclinical success and clinical outcomes. Future optimization will depend on integrating high-quality experimental datasets with computational models, improving algorithms to account for RNA secondary structures and modifications, and applying machine-learning approaches trained on experimentally verified interactions. Collectively, overcoming these barriers is critical for improving the reliability, safety, and bench-to-bedside success of RNA therapeutics^210^.

## Future perspectives and outlook

Looking ahead, RNA-based therapeutics are expected to grow well beyond traditional approaches such as mRNA, siRNA, and CRISPR. New discoveries in RNA biology have revealed many types of non-coding RNAs (ncRNAs) that help control gene activity, chromatin structure, and RNA modifications within cells. These newly recognized RNA molecules not only deepen our understanding of how genes are regulated but also show great potential as future therapeutic tools and disease biomarkers. Including these ncRNAs in the field of RNA medicine will be an important step toward developing more comprehensive and versatile treatments.

### Emerging non-coding RNA therapeutics: expanding the RNA frontier

The landscape of RNA therapeutics is rapidly broadening beyond canonical messenger RNA (mRNA) and RNA interference (RNAi) approaches to encompass a diverse spectrum of non-coding RNAs (ncRNAs) with distinctive structural and regulatory functions. These molecules, tRNA-derived fragments (tRFs), long non-coding RNAs (lncRNAs), small nucleolar RNAs (snoRNAs), and circular RNAs (circRNAs), are reshaping our understanding of gene regulation, epigenetic control, and post-transcriptional processing. With advances in high-throughput sequencing, structural RNA mapping, and chemical modification technologies, these ncRNAs are now recognized as key regulators in health and disease, offering promising new modalities for precision therapeutics, disease biomarkers, and RNA-based drug discovery^212^.

### tRNA-derived fragments (tRFs): from translational control to therapeutic modulation

Once considered random degradation products, tRNA-derived fragments (tRFs) have emerged as small, functionally active RNAs (14–50 nucleotides) with roles in translation regulation, stress adaptation, and signaling. They are categorized as 5′-tRFs, 3′-tRFs, internal tRFs, and tRNA halves (tiRNAs), each generated by precise cleavage of mature or precursor tRNAs. These fragments associate with Argonaute and YBX1 proteins to modulate mRNA stability and translation, while others interact with ribosomal subunits to fine-tune protein synthesis^213^.

Dysregulation of tRF expression has been implicated in tumorigenesis, neurodegeneration, and metabolic disorders. Functionally, tRFs act as molecular rheostats, capable of adjusting gene expression rather than completely silencing targets as siRNAs do. Chemically stabilized tRF mimics have shown antiproliferative and immunomodulatory activity in preclinical cancer models, whereas ASOs targeting oncogenic tRFs are being explored for glioblastoma and prostate cancer^214^. Due to their small size and inherent stability, tRF-based drugs may utilize existing delivery technologies such as GalNAc conjugation for liver targeting or LNPs for systemic administration. These properties make tRFs a unique bridge between classical RNA interference and emerging small RNA therapeutics^215^.

### Long non-coding RNAs (lncRNAs): master regulators and therapeutic targets

Long non-coding RNAs (lncRNAs), transcripts longer than 200 nucleotides with minimal coding potential, represent a highly diverse and functionally versatile class of ncRNAs. They orchestrate gene regulation through mechanisms including epigenetic remodeling, transcriptional interference, and post-transcriptional modulation. LncRNAs act as scaffolds for chromatin-modifying complexes, decoys for RNA-binding proteins, or competing endogenous RNAs (ceRNAs) that sequester microRNAs^216^. Aberrant lncRNA expression contributes to multiple disease pathologies, notably oncogenesis, cardiovascular dysfunction, and inflammatory disorders. Therapeutic inhibition of pathogenic lncRNAs such as *MALAT1*, *HOTAIR*, and *MIAT* using ASOs or locked nucleic acids (LNAs) has produced encouraging preclinical results. Conversely, synthetic lncRNA mimics are being engineered to function as molecular “sponges” for disease-associated miRNAs^217^.

Translational challenges persist, particularly with delivery and localization, as many lncRNAs exert their effects in the nucleus. Viral vectors, exosome-based carriers, and LNP formulations are under investigation for improving tissue specificity and nuclear targeting. Moreover, the capacity of lncRNAs to interact with chromatin regulators offers an exciting interface with CRISPR-based epigenome editing, suggesting potential for programmable, reversible modulation of gene expression^218^.

### Small nucleolar RNAs (snoRNAs): epitranscriptomic regulators in disease

Traditionally recognized for guiding 2′-O-methylation and pseudouridylation of ribosomal RNAs and small nuclear RNAs, small nucleolar RNAs (snoRNAs) are now being re-evaluated as multifaceted regulators of cell physiology. Beyond their canonical roles in ribosome biogenesis, snoRNAs contribute to metabolic control, immune signaling, and tumor progression^219^. Aberrant expression of snoRNAs and their host genes alters translational fidelity and ribosome specialization, thereby influencing the proteomic landscape of cancer and metabolic diseases. Therapeutically, snoRNA knockdown via ASOs or RNA interference can normalize dysregulated translation and restore homeostasis. Additionally, circulating snoRNAs exhibit remarkable stability, making them promising liquid biopsy biomarkers for early disease detection and monitoring. Emerging research highlights the epitranscriptomic dimension of snoRNA biology by modulating rRNA modifications, snoRNAs may indirectly reprogram global translation, providing a new layer of therapeutic intervention distinct from mRNA or siRNA approaches^220^.

### Circular RNAs (circRNAs): stable platforms for RNA therapy

Among the ncRNAs, circular RNAs (circRNAs) represent a particularly promising platform for next-generation RNA therapy. Generated by back-splicing of precursor mRNAs, circRNAs form covalently closed structures that confer exceptional nuclease resistance, extended intracellular half-life, and reduced innate immune activation^221^. CircRNAs function as miRNA sponges, protein scaffolds, and, in engineered systems, translation templates capable of producing therapeutic proteins. Synthetic circRNAs encoding antigens or enzymes have achieved sustained expression and potent immunogenicity in preclinical models, outperforming linear IVT mRNAs in durability and safety. Early experimental circRNA vaccines have induced long-lasting immune responses while minimizing inflammatory activation^222^.

Endogenous circRNAs such as *circZNF609* and *circHIPK3* regulate angiogenesis and neuronal differentiation, making them attractive targets for cardiovascular and neuroregenerative therapies. Challenges include scalable production, optimization of back-splicing efficiency, and precise expression control, but continued advances in circRNA design and delivery are rapidly overcoming these barriers. Together, circRNAs bridge the gap between transient mRNA therapy and permanent gene editing, offering durable yet controllable expression systems^222^.

### Translational opportunities and challenges

Collectively, the study of non-coding RNAs (ncRNAs) significantly broadens the therapeutic repertoire of RNA medicine by introducing novel mechanisms of action and molecular precision. Among these, tRNA-derived fragments (tRFs) provide fine-tuned regulation of translation and cellular stress pathways, while long non-coding RNAs (lncRNAs) enable modulation of chromatin structure and transcriptional activity through diverse epigenetic and post-transcriptional interactions. Small nucleolar RNAs (snoRNAs) function as epitranscriptomic regulators capable of adjusting translational outputs via RNA modification, and circular RNAs (circRNAs) offer stable, long-lasting expression vectors for sustained therapeutic protein production.

Despite these advances, several translational hurdles persist, including the need for tissue-specific and subcellular delivery, particularly for nuclear-acting RNAs, scalable and GMP-compliant manufacturing, reduction of off-target effects and immunogenicity, and the establishment of reliable biomarkers for patient stratification. Strategic integration of ncRNA modalities with existing delivery technologies, such as LNPs, GalNAc conjugates, exosomes, and viral vectors, in combination with orthogonal platforms like ASOs, CRISPR-based epigenetic editors, and small-molecule modulators, represents the most practical route toward clinical translation. As the field progresses, ncRNAs are expected not merely to complement existing RNA therapeutics but to serve as central components of a multi-layered RNA medicine framework, enabling precise and combinatorial interventions at the levels of sequence, structure, and RNA modification^223^.

## Conclusion and future prospects

Various RNA-based strategies have been employed in both experimental and clinical settings. Standard tools in cell and animal studies include commoditized ASOs, siRNAs, RNAi therapeutics, and mRNA vaccination. Several ASOs, miRNA, siRNA, and mRNA vaccines have received clinical approval. These approaches enable the modulation of specific mRNA expression and the suppression of RNA activity by selectively targeting designated RNA sequences. However, a significant barrier to the broader application of RNA-based therapies is the challenge of efficiently delivering these drugs to target organs and tissues outside the liver^224^. Moreover, the natural RNA processing mechanisms can diminish the efficacy of these strategies, challenges such as off-target interactions, sequence-related toxicity, and the risk of overstimulation^142^.

Chemical modification is a highly promising strategy for delivering RNA-based drugs. Modifying the nucleic acid backbone, ribose ring, and nucleobase enhances drug-like characteristics for better delivery. For instance, gapmer ASOs can be delivered to different tissues without the need for additional delivery agents due to their substantial chemical modifications. Presently, no extra delivery vehicles are used to administer eight of the ten oligonucleotide therapies that have been approved. On the other hand, some artificial nucleotides, such as LNA-modified nucleic acids, can be dangerous and lead to significant hepatotoxicity^225^. To address this, bioengineered RNAi agents (BERAs) with minimal post-transcriptional modifications show great potential^226^. Developing nanocarriers for RNA drug delivery has greatly enhanced liver-specific transport; however, more research is needed for systemic nanomedicines targeting non-liver tissues. Progress in imaging technologies, like electron microscopy and super-resolution fluorescence microscopy, combined with omics-based strategies, has enabled researchers to investigate intracellular delivery mechanisms. This has driven breakthroughs in nanoformulations and the strategic design of advanced delivery systems^227^.

Additionally, small-molecule-based therapies targeting RNA are often preferred. For example, Evrysdi (risdiplam) is the first orally bioavailable small-molecule inhibitor approved by the FDA that targets SMN2 pre-messenger RNA splicing explicitly^228,229^. It can be synthesized effectively and administered with ease. For additional guidance, reviews, and tutorials on creating small molecules that target RNA are readily available. The effectiveness of RNA medication delivery can be increased by combining RNA chemical changes with nanocarrier technologies. More RNA-based medicines for patient care will be developed due to ongoing research in RNA-based therapeutics, which includes both the utilization of RNA molecules as treatments and the targeting of RNA with small molecules.

## Data Availability

No primary research results, software, or code have been included, and no new data were generated or analyzed as part of this review.

## References

[1] Liu, M. et al. Landscape of small nucleic acid therapeutics: moving from the bench to the clinic as next-generation medicines. *Signal Transduct. Target. Ther.* **10**, 73 (2025). 40059188 10.1038/s41392-024-02112-8 PMC11891339

[2] Crick, F. H. On protein synthesis. *Symp. Soc. Exp. Biol.* **12**, 138–163 (1958). 13580867

[3] Brenner, S., Jacob, F. & Meselson, M. An unstable intermediate carrying information from genes to ribosomes for protein synthesis. *Nature* **190**, 576–581 (1961). 20446365 10.1038/190576a0

[4] Gros, F. et al. Unstable ribonucleic acid revealed by pulse labelling of *Escherichia coli*. *Nature* **190**, 581–585 (1961). 13708983 10.1038/190581a0

[5] Rich, A. & Davies, D. R. A new two stranded helical structure: polyadenylic acid and polyuridylic acid. *J. Am. Chem. Soc.* **78**, 3548–3549 (1956).

[6] Lee, R. C., Feinbaum, R. L. & Ambros, V. The *C. elegans* heterochronic gene lin-4 encodes small RNAs with antisense complementarity to lin-14. *Cell* **75**, 843–854 (1993). 8252621 10.1016/0092-8674(93)90529-y

[7] Fire, A. et al. Potent and specific genetic interference by double-stranded RNA in *Caenorhabditis elegans*. *Nature* **391**, 806–811 (1998). 9486653 10.1038/35888

[8] Zamecnik, P. C. & Stephenson, M. L. Inhibition of Rous sarcoma virus replication and cell transformation by a specific oligodeoxynucleotide. *Proc. Natl. Acad. Sci. USA* **75**, 280–284 (1978). 75545 10.1073/pnas.75.1.280 PMC411230

[9] Roehr, B. Fomivirsen approved for CMV retinitis. *J. Int. Assoc. Physicians AIDS Care* **4**, 14–16 (1998). 11365956

[10] Berget, S. M., Moore, C. & Sharp, P. A. Spliced segments at the 5’ terminus of adenovirus 2 late mRNA. *Proc. Natl. Acad. Sci. USA* **74**, 3171–3175 (1977). 269380 10.1073/pnas.74.8.3171 PMC431482

[11] Chow, L. T., Gelinas, R. E., Broker, T. R. & Roberts, R. J. An amazing sequence arrangement at the 5’ ends of adenovirus 2 messenger RNA. *Cell* **12**, 1–8 (1977). 902310 10.1016/0092-8674(77)90180-5

[12] Scotti, M. M. & Swanson, M. S. RNA mis-splicing in disease. *Nat. Rev. Genet.* **17**, 19–32 (2016). 26593421 10.1038/nrg.2015.3 PMC5993438

[13] Baranello, G. et al. Risdiplam in type 1 spinal muscular atrophy. *N. Engl. J. Med.* **384**, 915–923 (2021). 33626251 10.1056/NEJMoa2009965

[14] Finkel, R. S. et al. Nusinersen versus sham control in infantile-onset spinal muscular atrophy. *N. Engl. J. Med.* **377**, 1723–1732 (2017). 29091570 10.1056/NEJMoa1702752

[15] Dominski, Z. & Kole, R. Restoration of correct splicing in thalassemic pre-mRNA by antisense oligonucleotides. *Proc. Natl. Acad. Sci. USA* **90**, 8673–8677 (1993). 8378346 10.1073/pnas.90.18.8673 PMC47420

[16] McCaffrey, A. P. et al. RNA interference in adult mice. *Nature* **418**, 38–39 (2002). 12097900 10.1038/418038a

[17] Davis, M. E. et al. Evidence of RNAi in humans from systemically administered siRNA via targeted nanoparticles. *Nature* **464**, 1067–1070 (2010). 20305636 10.1038/nature08956 PMC2855406

[18] Adams, D. et al. Patisiran, an RNAi therapeutic, for hereditary transthyretin amyloidosis. *N. Engl. J. Med.* **379**, 11–21 (2018). 29972753 10.1056/NEJMoa1716153

[19] Baltimore, D. & Franklin, R. M. Preliminary data on a virus-specific enzyme system responsible for the synthesis of viral RNA. *Biochem. Biophys. Res. Commun.* **9**, 388–392 (1962). 13966258 10.1016/0006-291x(62)90021-9

[20] Bloom, K., van den Berg, F. & Arbuthnot, P. Self-amplifying RNA vaccines for infectious diseases. *Gene Ther.* **28**, 117–129 (2021). 33093657 10.1038/s41434-020-00204-y PMC7580817

[21] Wolff, J. A. et al. Direct gene transfer into mouse muscle in vivo. *Science* **247**, 1465–1468 (1990). 1690918 10.1126/science.1690918

[22] Martinon, F. et al. Induction of virus-specific cytotoxic T lymphocytes in vivo by liposome-entrapped mRNA. *Eur. J. Immunol.* **23**, 1719–1722 (1993). 8325342 10.1002/eji.1830230749

[23] Conry, R. M. et al. Characterization of a messenger RNA polynucleotide vaccine vector. *Cancer Res*. **55**, 1397–1400 (1995). 7882341

[24] Weide, B. et al. Results of the first phase I/II clinical vaccination trial with direct injection of mRNA. *J. Immunother.* **31**, 180–188 (2008). 18481387 10.1097/CJI.0b013e31815ce501

[25] Weide, B. et al. Direct injection of protamine-protected mRNA: results of a phase 1/2 vaccination trial in metastatic melanoma patients. *J. Immunother.* **32**, 498–507 (2009). 19609242 10.1097/CJI.0b013e3181a00068

[26] Alberer, M. et al. Safety and immunogenicity of a mRNA rabies vaccine in healthy adults: an open-label, non-randomised, prospective, first-in-human phase 1 clinical trial. *Lancet* **390**, 1511–1520 (2017). 28754494 10.1016/S0140-6736(17)31665-3

[27] Sparmann, A. & Vogel, J. RNA-based medicine: from molecular mechanisms to therapy. *EMBO J.* **42**, e114760 (2023). 37728251 10.15252/embj.2023114760 PMC10620767

[28] Stephenson, M. L. & Zamecnik, P. C. Inhibition of Rous sarcoma viral RNA translation by a specific oligodeoxyribonucleotide. *Proc. Natl. Acad. Sci. USA* **75**, 285–288 (1978). 75546 10.1073/pnas.75.1.285 PMC411231

[29] de Smet, M. D., Meenken, C. & van den Horn, G. J. Fomivirsen—a phosphorothioate oligonucleotide for the treatment of CMV retinitis. *Ocul. Immunol. Inflamm.* **7**, 189–198 (1999). 10611727 10.1076/ocii.7.3.189.4007

[30] Group, V. S. A randomized controlled clinical trial of intravitreous fomivirsen for treatment of newly diagnosed peripheral cytomegalovirus retinitis in patients with AIDS. *Am. J. Ophthalmol.* **133**, 467–474 (2002). 11931780 10.1016/s0002-9394(02)01327-2

[31] Group, V. S. Randomized dose-comparison studies of intravitreous fomivirsen for treatment of cytomegalovirus retinitis that has reactivated or is persistently active despite other therapies in patients with AIDS. *Am. J. Ophthalmol.* **133**, 475–483 (2002). 11931781 10.1016/s0002-9394(02)01326-0

[32] Dhuri, K. et al. Antisense oligonucleotides: an emerging area in drug discovery and development. *J Clin. Med*. **9**, 2004 (2020). 10.3390/jcm9062004 PMC7355792 32604776

[33] Crooke, S. T. E. *Antisense Drug Technology: Principles, Strategies, and Applications* 2nd edn (CRC Press, 2007).

[34] Crooke, S. T., Witztum, J. L., Bennett, C. F. & Baker, B. F. RNA-targeted therapeutics. *Cell Metab.* **27**, 714–739 (2018). 29617640 10.1016/j.cmet.2018.03.004

[35] Crooke, S. T., Baker, B. F., Crooke, R. M. & Liang, X. H. Antisense technology: an overview and prospectus. *Nat. Rev. Drug Discov.* **20**, 427–453 (2021). 33762737 10.1038/s41573-021-00162-z

[36] Liang, X. H., Sun, H., Nichols, J. G. & Crooke, S. T. RNase H1-dependent antisense oligonucleotides are robustly active in directing RNA cleavage in both the cytoplasm and the nucleus. *Mol. Ther.* **25**, 2075–2092 (2017). 28663102 10.1016/j.ymthe.2017.06.002 PMC5589097

[37] Mulhbacher, J., St-Pierre, P. & Lafontaine, D. A. Therapeutic applications of ribozymes and riboswitches. *Curr. Opin. Pharm.* **10**, 551–556 (2010). 10.1016/j.coph.2010.07.002 20685165

[38] Sullivan, S. M. Development of ribozymes for gene therapy. *J. Invest. Dermatol.* **103**, 85s–89s (1994). 7963690 10.1038/jid.1994.15

[39] Ertural, B., Çiçek, B. N. & Kurnaz, I. A. RNA therapeutics: focus on antisense oligonucleotides in the nervous system. *Biomol. Ther.* **33**, 572–581 (2025). 10.4062/biomolther.2025.022 PMC12215037 40534528

[40] Rigo, F., Hua, Y., Krainer, A. R. & Bennett, C. F. Antisense-based therapy for the treatment of spinal muscular atrophy. *J. Cell Biol.* **199**, 21–25 (2012). 23027901 10.1083/jcb.201207087 PMC3461520

[41] Liang, X. H. et al. Antisense oligonucleotides targeting translation inhibitory elements in 5’ UTRs can selectively increase protein levels. *Nucleic Acids Res.* **45**, 9528–9546 (2017). 28934489 10.1093/nar/gkx632 PMC5766168

[42] Melton, D. A. Injected anti-sense RNAs specifically block messenger RNA translation in vivo. *Proc. Natl. Acad. Sci. USA* **82**, 144–148 (1985). 3855537 10.1073/pnas.82.1.144 PMC396988

[43] Iversen, P. L., Arora, V., Acker, A. J., Mason, D. H. & Devi, G. R. Efficacy of antisense morpholino oligomer targeted to c-myc in prostate cancer xenograft murine model and a Phase I safety study in humans. *Clin. Cancer Res.* **9**, 2510–2519 (2003). 12855625

[44] Baker, B. F. et al. Oligonucleotide-europium complex conjugate designed to cleave the 5’ cap structure of the ICAM-1 transcript potentiates antisense activity in cells. *Nucleic Acids Res.* **27**, 1547–1551 (1999). 10037819 10.1093/nar/27.6.1547 PMC148351

[45] Vickers, T. A., Wyatt, J. R., Burckin, T., Bennett, C. F. & Freier, S. M. Fully modified 2’ MOE oligonucleotides redirect polyadenylation. *Nucleic Acids Res.* **29**, 1293–1299 (2001). 11238995 10.1093/nar/29.6.1293 PMC29745

[46] Hua, Y., Vickers, T. A., Baker, B. F., Bennett, C. F. & Krainer, A. R. Enhancement of SMN2 exon 7 inclusion by antisense oligonucleotides targeting the exon. *PLoS Biol.* **5**, e73 (2007). 17355180 10.1371/journal.pbio.0050073 PMC1820610

[47] Bennett, C. F. Therapeutic antisense oligonucleotides are coming of age. *Annu. Rev. Med.* **70**, 307–321 (2019). 30691367 10.1146/annurev-med-041217-010829

[48] Hodges, D. & Crooke, S. T. Inhibition of splicing of wild-type and mutated luciferase-adenovirus pre-mRNAs by antisense oligonucleotides. *Mol. Pharm.* **48**, 905–918 (1995). 7476922

[49] Charleston, J. S. et al. Eteplirsen treatment for Duchenne muscular dystrophy: exon skipping and dystrophin production. *Neurology* **90**, e2146–e2154 (2018). 29752304 10.1212/WNL.0000000000005680

[50] Darras, B. T. et al. An integrated safety analysis of infants and children with symptomatic spinal muscular atrophy (SMA) treated with nusinersen in seven clinical trials. *CNS Drugs* **33**, 919–932 (2019). 31420846 10.1007/s40263-019-00656-w PMC6776494

[51] Hua, Y., Vickers, T. A., Okunola, H. L., Bennett, C. F. & Krainer, A. R. Antisense masking of an hnRNP A1/A2 intronic splicing silencer corrects SMN2 splicing in transgenic mice. *Am. J. Hum. Genet.* **82**, 834–848 (2008). 18371932 10.1016/j.ajhg.2008.01.014 PMC2427210

[52] Ward, A. J., Norrbom, M., Chun, S., Bennett, C. F. & Rigo, F. Nonsense-mediated decay as a terminating mechanism for antisense oligonucleotides. *Nucleic Acids Res.* **42**, 5871–5879 (2014). 24589581 10.1093/nar/gku184 PMC4027159

[53] Liang, X. H. et al. Translation efficiency of mRNAs is increased by antisense oligonucleotides targeting upstream open reading frames. *Nat. Biotechnol.* **34**, 875–880 (2016). 27398791 10.1038/nbt.3589

[54] Liang, X. H., Nichols, J. G., Sun, H. & Crooke, S. T. Translation can affect the antisense activity of RNase H1-dependent oligonucleotides targeting mRNAs. *Nucleic Acids Res*. **46**, 293–313 (2018). 29165591 10.1093/nar/gkx1174 PMC5758896

[55] Sands, H. et al. Biodistribution and metabolism of internally 3H-labeled oligonucleotides. I. Comparison of a phosphodiester and a phosphorothioate. *Mol. Pharm.* **45**, 932–943 (1994). 8190109

[56] Dowdy, S. F. Endosomal escape of RNA therapeutics: how do we solve this rate-limiting problem? *RNA* **29**, 396–401 (2023). 36669888 10.1261/rna.079507.122 PMC10019367

[57] Chery, J. RNA therapeutics: RNAi and antisense mechanisms and clinical applications. *Postdoc J.* **4**, 35–50 (2016). 27570789 10.14304/surya.jpr.v4n7.5 PMC4995773

[58] Gagliardi, M. & Ashizawa, A. T. The challenges and strategies of antisense oligonucleotide drug delivery. *Biomedicines* **9**, 433 (2021). 10.3390/biomedicines9040433 PMC8072990 33923688

[59] Dalpke, A. & Helm, M. RNA mediated Toll-like receptor stimulation in health and disease. *RNA Biol.* **9**, 828–842 (2012). 22617878 10.4161/rna.20206 PMC3495747

[60] Chen, Y. G. & Hur, S. Cellular origins of dsRNA, their recognition and consequences. *Nat. Rev. Mol. Cell Biol.* **23**, 286–301 (2022). 34815573 10.1038/s41580-021-00430-1 PMC8969093

[61] Khvorova, A. & Watts, J. K. The chemical evolution of oligonucleotide therapies of clinical utility. *Nat. Biotechnol.* **35**, 238–248 (2017). 28244990 10.1038/nbt.3765 PMC5517098

[62] Djuranovic, S., Nahvi, A. & Green, R. miRNA-mediated gene silencing by translational repression followed by mRNA deadenylation and decay. *Science* **336**, 237–240 (2012). 22499947 10.1126/science.1215691 PMC3971879

[63] Hutvagner, G. & Zamore, P. D. A microRNA in a multiple-turnover RNAi enzyme complex. *Science* **297**, 2056–2060 (2002). 12154197 10.1126/science.1073827

[64] Sano, M. et al. Effect of asymmetric terminal structures of short RNA duplexes on the RNA interference activity and strand selection. *Nucleic Acids Res.* **36**, 5812–5821 (2008). 18782830 10.1093/nar/gkn584 PMC2566866

[65] Varley, A. J. & Desaulniers, J.-P. Chemical strategies for strand selection in short-interfering RNAs. *RSC Adv.* **11**, 2415–2426 (2021). 35424193 10.1039/d0ra07747j PMC8693850

[66] Snead, N. M. et al. Molecular basis for improved gene silencing by Dicer substrate interfering RNA compared with other siRNA variants. *Nucleic Acids Res.* **41**, 6209–6221 (2013). 23620279 10.1093/nar/gkt200 PMC3695504

[67] Cuellar, T. L. et al. Systematic evaluation of antibody-mediated siRNA delivery using an industrial platform of THIOMAB-siRNA conjugates. *Nucleic Acids Res.* **43**, 1189–1203 (2015). 25550431 10.1093/nar/gku1362 PMC4333408

[68] Dugal-Tessier, J., Thirumalairajan, S. & Jain, N. Antibody-oligonucleotide conjugates: a twist to antibody-drug conjugates. *J Clin Med.* **10**, 838 (2021). 10.3390/jcm10040838 PMC7922418 33670689

[69] Malecova, B. et al. Targeted tissue delivery of RNA therapeutics using antibody-oligonucleotide conjugates (AOCs). *Nucleic Acids Res*. **51**, 5901–5910 (2023). 37224533 10.1093/nar/gkad415 PMC10325888

[70] Klabenkova, K., Fokina, A. & Stetsenko, D. Chemistry of peptide-oligonucleotide conjugates: a review. *Molecules* **26**, 5420 (2021). 10.3390/molecules26175420 PMC8434111 34500849

[71] Biscans, A. et al. Diverse lipid conjugates for functional extra-hepatic siRNA delivery in vivo. *Nucleic Acids Res.* **47**, 1082–1096 (2019). 30544191 10.1093/nar/gky1239 PMC6379722

[72] Brown, K. M. et al. Expanding RNAi therapeutics to extrahepatic tissues with lipophilic conjugates. *Nat. Biotechnol.* **40**, 1500–1508 (2022). 35654979 10.1038/s41587-022-01334-x

[73] Alterman, J. F. et al. A divalent siRNA chemical scaffold for potent and sustained modulation of gene expression throughout the central nervous system. *Nat. Biotechnol.* **37**, 884–894 (2019). 31375812 10.1038/s41587-019-0205-0 PMC6879195

[74] Han, S. P. et al. Programmable siRNA pro-drugs that activate RNAi activity in response to specific cellular RNA biomarkers. *Mol. Ther. Nucleic Acids* **27**, 797–809 (2022). 35116191 10.1016/j.omtn.2021.12.039 PMC8789579

[75] Rupaimoole, R. & Slack, F. J. MicroRNA therapeutics: towards a new era for the management of cancer and other diseases. *Nat. Rev. Drug Discov.* **16**, 203–222 (2017). 28209991 10.1038/nrd.2016.246

[76] Lanford, R. E. et al. Therapeutic silencing of microRNA-122 in primates with chronic hepatitis C virus infection. *Science* **327**, 198–201 (2010). 19965718 10.1126/science.1178178 PMC3436126

[77] Janssen, H. L. et al. Treatment of HCV infection by targeting microRNA. *N. Engl. J. Med.* **368**, 1685–1694 (2013). 23534542 10.1056/NEJMoa1209026

[78] Davis, S. M. et al. Chemical optimization of siRNA for safe and efficient silencing of placental sFLT1. *Mol. Ther. Nucleic Acids* **29**, 135–149 (2022). 35847173 10.1016/j.omtn.2022.06.009 PMC9263991

[79] Sahin, U., Karikó, K. & Türeci, Ö. mRNA-based therapeutics-developing a new class of drugs. *Nat. Rev. Drug Discov.* **13**, 759–780 (2014). 25233993 10.1038/nrd4278

[80] Karikó, K., Muramatsu, H., Ludwig, J. & Weissman, D. Generating the optimal mRNA for therapy: HPLC purification eliminates immune activation and improves translation of nucleoside-modified, protein-encoding mRNA. *Nucleic Acids Res*. **39**, e142 (2011). 21890902 10.1093/nar/gkr695 PMC3241667

[81] Chaudhary, N., Weissman, D. & Whitehead, K. A. mRNA vaccines for infectious diseases: principles, delivery and clinical translation. *Nat. Rev. Drug Discov.* **20**, 817–838 (2021). 34433919 10.1038/s41573-021-00283-5 PMC8386155

[82] Beck, J. D. et al. mRNA therapeutics in cancer immunotherapy. *Mol. Cancer* **20**, 69 (2021). 33858437 10.1186/s12943-021-01348-0 PMC8047518

[83] Polack, F. P. et al. Safety and efficacy of the BNT162b2 mRNA Covid-19 vaccine. *N. Engl. J. Med.* **383**, 2603–2615 (2020). 33301246 10.1056/NEJMoa2034577 PMC7745181

[84] Baden, L. R. et al. Efficacy and safety of the mRNA-1273 SARS-CoV-2 vaccine. *N. Engl. J. Med.* **384**, 403–416 (2021). 33378609 10.1056/NEJMoa2035389 PMC7787219

[85] Sahin, U. & Tureci, O. Personalized vaccines for cancer immunotherapy. *Science* **359**, 1355–1360 (2018). 29567706 10.1126/science.aar7112

[86] Rojas, L. A. et al. Personalized RNA neoantigen vaccines stimulate T cells in pancreatic cancer. *Nature* **618**, 144–150 (2023). 37165196 10.1038/s41586-023-06063-y PMC10171177

[87] Dolgin, E. Personalized cancer vaccines pass first major clinical test. *Nat. Rev. Drug Discov.* **22**, 607–609 (2023). 37438497 10.1038/d41573-023-00118-5

[88] Lang, F., Schrors, B., Lower, M., Tureci, O. & Sahin, U. Identification of neoantigens for individualized therapeutic cancer vaccines. *Nat. Rev. Drug Discov.* **21**, 261–282 (2022). 35105974 10.1038/s41573-021-00387-y PMC7612664

[89] Krienke, C. et al. A noninflammatory mRNA vaccine for treatment of experimental autoimmune encephalomyelitis. *Science* **371**, 145–153 (2021). 33414215 10.1126/science.aay3638

[90] Anttila, V. et al. Synthetic mRNA encoding VEGF-A in patients undergoing coronary artery bypass grafting: design of a phase 2a clinical trial. *Mol. Ther. Methods Clin. Dev.* **18**, 464–472 (2020). 32728595 10.1016/j.omtm.2020.05.030 PMC7369517

[91] Hille, F. et al. The biology of CRISPR-Cas: backward and forward. *Cell* **172**, 1239–1259 (2018). 29522745 10.1016/j.cell.2017.11.032

[92] Gasiunas, G., Barrangou, R., Horvath, P. & Siksnys, V. Cas9-crRNA ribonucleoprotein complex mediates specific DNA cleavage for adaptive immunity in bacteria. *Proc. Natl. Acad. Sci. USA* **109**, E2579–E2586 (2012). 22949671 10.1073/pnas.1208507109 PMC3465414

[93] Jinek, M. et al. A programmable dual-RNA-guided DNA endonuclease in adaptive bacterial immunity. *Science* **337**, 816–821 (2012). 22745249 10.1126/science.1225829 PMC6286148

[94] Doudna, J. A. & Charpentier, E. Genome editing. The new frontier of genome engineering with CRISPR-Cas9. *Science* **346**, 1258096 (2014). 25430774 10.1126/science.1258096

[95] Wang, F. et al. Advances in CRISPR-Cas systems for RNA targeting, tracking and editing. *Biotechnol. Adv.* **37**, 708–729 (2019). 30926472 10.1016/j.biotechadv.2019.03.016

[96] O’Connell, M. R. et al. Programmable RNA recognition and cleavage by CRISPR/Cas9. *Nature* **516**, 263–266 (2014). 25274302 10.1038/nature13769 PMC4268322

[97] Strutt, S. C., Torrez, R. M., Kaya, E., Negrete, O. A. & Doudna, J. A. RNA-dependent RNA targeting by CRISPR-Cas9. *Elife* **7**, e32724 (2018). 10.7554/eLife.32724 PMC5796797 29303478

[98] Konermann, S. et al. Transcriptome engineering with RNA-targeting type VI-D CRISPR effectors. *Cell* **173**, 665–676.e614 (2018). 29551272 10.1016/j.cell.2018.02.033 PMC5910255

[99] Cox, D. B. T. et al. RNA editing with CRISPR-Cas13. *Science* **358**, 1019–1027 (2017). 29070703 10.1126/science.aaq0180 PMC5793859

[100] Chapman, J. R., Taylor, M. R. & Boulton, S. J. Playing the end game: DNA double-strand break repair pathway choice. *Mol. Cell* **47**, 497–510 (2012). 22920291 10.1016/j.molcel.2012.07.029

[101] Yeh, C. D., Richardson, C. D. & Corn, J. E. Advances in genome editing through control of DNA repair pathways. *Nat. Cell Biol.* **21**, 1468–1478 (2019). 31792376 10.1038/s41556-019-0425-z

[102] Uddin, F., Rudin, C. M. & Sen, T. CRISPR gene therapy: applications, limitations, and implications for the future. *Front. Oncol.* **10**, 1387 (2020). 32850447 10.3389/fonc.2020.01387 PMC7427626

[103] Zhang, W. et al. Lipid carriers for mRNA delivery. *Acta Pharm. Sin. B* **13**, 4105–4126 (2023). 37799378 10.1016/j.apsb.2022.11.026 PMC10547918

[104] Azeez, S. S., Hamad, R. S., Hamad, B. K., Shekha, M. S. & Bergsten, P. Advances in CRISPR-Cas technology and its applications: revolutionising precision medicine. *Front. Genome Ed.* **6**, 1509924 (2024). 39726634 10.3389/fgeed.2024.1509924 PMC11669675

[105] Eftekhari, Z. et al. Advancements and challenges in mRNA and ribonucleoprotein-based therapies: from delivery systems to clinical applications. *Mol. Ther. Nucl. Acids* **35**, 102313 (2024). 10.1016/j.omtn.2024.102313 PMC11402252 39281702

[106] Thakral, S., Purohit, P., Mishra, R., Gupta, V. & Setia, P. The impact of RNA stability and degradation in different tissues to the determination of post-mortem interval: a systematic review. *Forensic Sci. Int.* **349**, 111772 (2023). 37450949 10.1016/j.forsciint.2023.111772

[107] Cheng, F. et al. Research advances on the stability of mRNA vaccines. *Viruses* **15**, 668 (2023). 10.3390/v15030668 PMC10051489 36992377

[108] Zhang, H. et al. Algorithm for optimized mRNA design improves stability and immunogenicity. *Nature* **621**, 396–403 (2023). 37130545 10.1038/s41586-023-06127-z PMC10499610

[109] Abaza, T., Mohamed, E. E. & Zaky, M. Y. Lipid nanoparticles: a promising tool for nucleic acid delivery in cancer immunotherapy. *Med. Oncol.* **42**, 409 (2025). 40768059 10.1007/s12032-025-02939-3 PMC12328551

[110] Zhang, Y. et al. EnhanCed Hydrophilicity And Antifouling Performance of PEG with sulfoxide-containing side chains for nanomedicine applications. *Polym. Sci. Technol.* **1**, 640–650 (2025). 41019823 10.1021/polymscitech.5c00084 PMC12462242

[111] Cong, X. et al. Nanocarriers for targeted drug delivery in the vascular system: focus on endothelium. *J. Nanobiotechnol.* **22**, 620 (2024). 10.1186/s12951-024-02892-9 PMC11470712 39396002

[112] Schlich, M. et al. Cytosolic delivery of nucleic acids: the case of ionizable lipid nanoparticles. *Bioeng. Transl. Med.* **6**, e10213 (2021). 33786376 10.1002/btm2.10213 PMC7995196

[113] Ruseska, I. & Zimmer, A. Internalization mechanisms of cell-penetrating peptides. *Beilstein J. Nanotechnol.* **11**, 101–123 (2020). 31976201 10.3762/bjnano.11.10 PMC6964662

[114] Zheng, J., Sun, Y., Shen, Y. & Zhou, Z. Surface engineering of nanoparticles for precision medicine. *Precis. Med. Eng.* **2**, 100037 (2025).

[115] Lin, L., Su, K., Cheng, Q. & Liu, S. Targeting materials and strategies for RNA delivery. *Theranostics* **13**, 4667–4693 (2023). 37649616 10.7150/thno.87316 PMC10465230

[116] Chehelgerdi, M. et al. Progressing nanotechnology to improve targeted cancer treatment: overcoming hurdles in its clinical implementation. *Mol. Cancer* **22**, 169 (2023). 37814270 10.1186/s12943-023-01865-0 PMC10561438

[117] Ramírez-Cortés, F. & Ménová, P. Hepatocyte targeting via the asialoglycoprotein receptor. *RSC Med. Chem.* **16**, 525–544 (2025). 39628900 10.1039/d4md00652f PMC11609720

[118] Chen, Y., Lin, J., Zhao, Y., Ma, X. & Yi, H. Toll-like receptor 3 (TLR3) regulation mechanisms and roles in antiviral innate immune responses. *J. Zhejiang Univ. Sci. B* **22**, 609–632 (2021). 34414698 10.1631/jzus.B2000808 PMC8377577

[119] Li, D. & Wu, M. Pattern recognition receptors in health and diseases. *Signal Transduct. Target. Ther.* **6**, 291 (2021). 34344870 10.1038/s41392-021-00687-0 PMC8333067

[120] Wu, S., Liu, C., Bai, S., Lu, Z. & Liu, G. Broadening the horizons of RNA delivery strategies in cancer therapy. *Bioengineering* **9**, 576 (2022). 10.3390/bioengineering9100576 PMC9598637 36290544

[121] Ren, Y. et al. Impact of ionizable lipid type on the pharmacokinetics and biodistribution of mRNA-lipid nanoparticles after intravenous and subcutaneous injection. *J. Control Release* **384**, 113945 (2025). 40505892 10.1016/j.jconrel.2025.113945

[122] Zhao, Y. et al. Replacing cholesterol and PEGylated lipids with zwitterionic ionizable lipids in LNPs for spleen-specific mRNA translation. *Sci. Adv.* **11**, eady6460 (2025). 41061074 10.1126/sciadv.ady6460 PMC12506999

[123] Balwani, M. et al. Phase 3 trial of RNAi therapeutic givosiran for acute intermittent porphyria. *N. Engl. J. Med*. **382**, 2289–2301 (2020). 32521132 10.1056/NEJMoa1913147

[124] Ray, K. K. et al. Two phase 3 trials of inclisiran in patients with elevated LDL cholesterol. *N. Engl. J. Med*. **382**, 1507–1519 (2020). 32187462 10.1056/NEJMoa1912387

[125] Anttila, V. et al. Direct intramyocardial injection of VEGF mRNA in patients undergoing coronary artery bypass grafting. *Mol. Ther.* **31**, 866–874 (2023). 36528793 10.1016/j.ymthe.2022.11.017 PMC10014220

[126] Weber, J. S. et al. Individualised neoantigen therapy mRNA-4157 (V940) plus pembrolizumab versus pembrolizumab monotherapy in resected melanoma (KEYNOTE-942): a randomised, phase 2b study. *Lancet* **403**, 632–644 (2024). 38246194 10.1016/S0140-6736(23)02268-7

[127] Song, Y. et al. RNATACs: multispecific small molecules targeting RNA by induced proximity. *Cell Chem. Biol.* **31**, 1101–1117 (2024). 38876100 10.1016/j.chembiol.2024.05.006

[128] Rhodes, C., Balaratnam, S., Yazdani, K., Seshadri, S. & Schneekloth, J. S. Targeting RNA-protein interactions with small molecules: promise and therapeutic potential. *Med. Chem. Res.* **33**, 2050–2065 (2024).

[129] Cetin, B., Erendor, F., Eksi, Y. E., Sanlioglu, A. D. & Sanlioglu, S. Advancing CRISPR genome editing into gene therapy clinical trials: progress and future prospects. *Expert Rev. Mol. Med.* **27**, e16 (2025). 40160040 10.1017/erm.2025.10 PMC12094669

[130] Paunovska, K., Loughrey, D. & Dahlman, J. E. Drug delivery systems for RNA therapeutics. *Nat. Rev. Genet.* **23**, 265–280 (2022). 34983972 10.1038/s41576-021-00439-4 PMC8724758

[131] Kim, E. H. et al. Lipid nanoparticle-mediated RNA delivery for immune cell modulation. *Eur. J. Immunol.* **54**, e2451008 (2024). 39279550 10.1002/eji.202451008 PMC11628889

[132] Qin, S. et al. mRNA-based therapeutics: powerful and versatile tools to combat diseases. *Signal Transduct. Target. Ther.* **7**, 166 (2022). 35597779 10.1038/s41392-022-01007-w PMC9123296

[133] Kulkarni, J. A., Cullis, P. R. & van der Meel, R. Lipid nanoparticles enabling gene therapies: from concepts to clinical utility. *Nucleic Acid Ther.* **28**, 146–157 (2018). 29683383 10.1089/nat.2018.0721

[134] Yang, W., Mixich, L., Boonstra, E. & Cabral, H. Polymer-based mRNA delivery strategies for advanced therapies. *Adv. Health. Mater.* **12**, e2202688 (2023). 10.1002/adhm.202202688 PMC11469255 36785927

[135] Chen, J., Zhu, D., Liu, X. & Peng, L. Amphiphilic dendrimer vectors for RNA delivery: state-of-the-art and future. *Perspect. Acc. Mater. Res.* **3**, 484–497 (2022). 10.1021/accountsmr.1c00272 PMC9245573 35782755

[136] Esrick, E. B. et al. Post-transcriptional genetic silencing of BCL11A to treat sickle cell disease. *N. Engl. J. Med.* **384**, 205–215 (2021). 33283990 10.1056/NEJMoa2029392 PMC7962145

[137] Nguyen, G. N. et al. A long-term study of AAV gene therapy in dogs with hemophilia A identifies clonal expansions of transduced liver cells. *Nat. Biotechnol.* **39**, 47–55 (2021). 33199875 10.1038/s41587-020-0741-7 PMC7855056

[138] Chandler, M., Panigaj, M., Rolband, L. A. & Afonin, K. A. Challenges to optimizing RNA nanostructures for large scale production and controlled therapeutic properties. *Nanomedicine* **15**, 1331–1340 (2020). 32452262 10.2217/nnm-2020-0034 PMC7304434

[139] Leborgne, C. et al. IgG-cleaving endopeptidase enables in vivo gene therapy in the presence of anti-AAV neutralizing antibodies. *Nat. Med.* **26**, 1096–1101 (2020). 32483358 10.1038/s41591-020-0911-7

[140] Bulcha, J. T., Wang, Y., Ma, H., Tai, P. W. L. & Gao, G. Viral vector platforms within the gene therapy landscape. *Signal Transduct. Target. Ther.* **6**, 53 (2021). 33558455 10.1038/s41392-021-00487-6 PMC7868676

[141] Bashor, C. J., Hilton, I. B., Bandukwala, H., Smith, D. M. & Veiseh, O. Engineering the next generation of cell-based therapeutics. *Nat. Rev. Drug Discov.* **21**, 655–675 (2022). 35637318 10.1038/s41573-022-00476-6 PMC9149674

[142] Zhu, Y., Zhu, L., Wang, X. & Jin, H. RNA-based therapeutics: an overview and prospectus. *Cell Death Dis.* **13**, 644 (2022). 35871216 10.1038/s41419-022-05075-2 PMC9308039

[143] Cheng, X. & Lee, R. J. The role of helper lipids in lipid nanoparticles (LNPs) designed for oligonucleotide delivery. *Adv. Drug Deliv. Rev.* **99**, 129–137 (2016). 26900977 10.1016/j.addr.2016.01.022

[144] Harvie, P., Wong, F. M. & Bally, M. B. Use of poly(ethylene glycol)-lipid conjugates to regulate the surface attributes and transfection activity of lipid-DNA particles. *J. Pharm. Sci.* **89**, 652–663 (2000). 10756331 10.1002/(SICI)1520-6017(200005)89:5<652::AID-JPS11>3.0.CO;2-H

[145] Patel, P., Ibrahim, N. M. & Cheng, K. The Importance of apparent pKa in the development of nanoparticles encapsulating siRNA and mRNA. *Trends Pharm. Sci.* **42**, 448–460 (2021). 33875229 10.1016/j.tips.2021.03.002 PMC8148296

[146] Cheng, Q. et al. Selective organ targeting (SORT) nanoparticles for tissue-specific mRNA delivery and CRISPR-Cas gene editing. *Nat. Nanotechnol.* **15**, 313–320 (2020). 32251383 10.1038/s41565-020-0669-6 PMC7735425

[147] Hajj, K. A. et al. A potent branched-tail lipid nanoparticle enables multiplexed mRNA delivery and gene editing in vivo. *Nano Lett.* **20**, 5167–5175 (2020). 32496069 10.1021/acs.nanolett.0c00596 PMC7781386

[148] Thompson, M. G. et al. Interim estimates of vaccine effectiveness of BNT162b2 and mRNA-1273 COVID-19 vaccines in preventing SARS-CoV-2 infection among health care personnel, first responders, and other essential and frontline workers—eight U.S. locations, December 2020–March 2021. *Morb. Mortal. Wkly. Rep.* **70**, 495–500 (2021). 10.15585/mmwr.mm7013e3 PMC8022879 33793460

[149] Dobrowolski, C., Paunovska, K., Hatit, M. Z. C., Lokugamage, M. P. & Dahlman, J. E. Therapeutic RNA delivery for COVID and other diseases. *Adv. Health. Mater.* **10**, e2002022 (2021). 10.1002/adhm.202002022 PMC7995096 33661555

[150] Herrera, M., Kim, J., Eygeris, Y., Jozic, A. & Sahay, G. Illuminating endosomal escape of polymorphic lipid nanoparticles that boost mRNA delivery. *Biomater. Sci.* **9**, 4289–4300 (2021). 33586742 10.1039/d0bm01947j PMC8769212

[151] Semple, S. C. et al. Rational design of cationic lipids for siRNA delivery. *Nat. Biotechnol.* **28**, 172–176 (2010). 20081866 10.1038/nbt.1602

[152] Rothgangl, T. et al. In vivo adenine base editing of PCSK9 in macaques reduces LDL cholesterol levels. *Nat. Biotechnol.* **39**, 949–957 (2021). 34012094 10.1038/s41587-021-00933-4 PMC8352781

[153] Crucho, C. I. C. & Barros, M. T. Polymeric nanoparticles: a study on the preparation variables and characterization methods. *Mater. Sci. Eng. C Mater. Biol. Appl* **80**, 771–784 (2017). 28866227 10.1016/j.msec.2017.06.004

[154] Harada-Shiba, M. et al. Polyion complex micelles as vectors in gene therapy-pharmacokinetics and in vivo gene transfer. *Gene Ther.* **9**, 407–414 (2002). 11960317 10.1038/sj.gt.3301665

[155] Xiao, B. et al. Combination therapy for ulcerative colitis: orally targeted nanoparticles prevent mucosal damage and relieve inflammation. *Theranostics* **6**, 2250–2266 (2016). 27924161 10.7150/thno.15710 PMC5135446

[156] Ewe, A. et al. Optimized polyethylenimine (PEI)-based nanoparticles for siRNA delivery, analyzed in vitro and in an ex vivo tumor tissue slice culture model. *Drug Deliv. Transl. Res.* **7**, 206–216 (2017). 27334279 10.1007/s13346-016-0306-y

[157] Breunig, M., Lungwitz, U., Liebl, R. & Goepferich, A. Breaking up the correlation between efficacy and toxicity for nonviral gene delivery. *Proc. Natl. Acad. Sci. USA* **104**, 14454–14459 (2007). 17726101 10.1073/pnas.0703882104 PMC1964826

[158] Tan, L. et al. Optimization of an mRNA vaccine assisted with cyclodextrin-polyethyleneimine conjugates. *Drug Deliv. Transl. Res.* **10**, 678–689 (2020). 32048201 10.1007/s13346-020-00725-4

[159] Xiang, J. J. et al. IONP-PLL: a novel non-viral vector for efficient gene delivery. *J. Gene Med.* **5**, 803–817 (2003). 12950071 10.1002/jgm.419

[160] Yin, H. et al. Non-viral vectors for gene-based therapy. *Nat. Rev. Genet*. **15**, 541–555 (2014). 25022906 10.1038/nrg3763

[161] Choi, J. et al. Nonviral polymeric nanoparticles for gene therapy in pediatric CNS malignancies. *Nanomedicine* **23**, 102115 (2020). 31655205 10.1016/j.nano.2019.102115 PMC7027378

[162] Green, J. J., Langer, R. & Anderson, D. G. A combinatorial polymer library approach yields insight into nonviral gene delivery. *Acc. Chem. Res*. **41**, 749–759 (2008). 18507402 10.1021/ar7002336 PMC3490629

[163] Vandenbroucke, R. E. et al. Prolonged gene silencing in hepatoma cells and primary hepatocytes after small interfering RNA delivery with biodegradable poly(beta-amino esters). *J. Gene Med.* **10**, 783–794 (2008). 18470950 10.1002/jgm.1202

[164] Anderson, D. G., Akinc, A., Hossain, N. & Langer, R. Structure/property studies of polymeric gene delivery using a library of poly(beta-amino esters). *Mol. Ther.* **11**, 426–434 (2005). 15727939 10.1016/j.ymthe.2004.11.015

[165] Altınoglu, S., Wang, M. & Xu, Q. Combinatorial library strategies for synthesis of cationic lipid-like nanoparticles and their potential medical applications. *Nanomedicine* **10**, 643–657 (2015). 25723096 10.2217/nnm.14.192

[166] Mastorakos, P. et al. Highly compacted biodegradable DNA nanoparticles capable of overcoming the mucus barrier for inhaled lung gene therapy. *Proc. Natl. Acad. Sci. USA* **112**, 8720–8725 (2015). 26124127 10.1073/pnas.1502281112 PMC4507234

[167] Kozielski, K. L. et al. Cancer-selective nanoparticles for combinatorial siRNA delivery to primary human GBM in vitro and in vivo. *Biomaterials* **209**, 79–87 (2019). 31026613 10.1016/j.biomaterials.2019.04.020 PMC7122460

[168] Blanchard, E. L. et al. Treatment of influenza and SARS-CoV-2 infections via mRNA-encoded Cas13a in rodents. *Nat. Biotechnol.* **39**, 717–726 (2021). 33536629 10.1038/s41587-021-00822-w

[169] Li, Z. et al. Lipid-polymer hybrid “particle-in-particle” nanostructure gene delivery platform explored for lyophilizable DNA and mRNA COVID-19 vaccines. *Adv. Funct. Mater.* **32**, 2204462 (2022). 35942271 10.1002/adfm.202204462 PMC9349454

[170] Sharma, A. R. et al. Recent advances of metal-based nanoparticles in nucleic acid delivery for therapeutic applications. *J. Nanobiotechnol.* **20**, 501 (2022). 10.1186/s12951-022-01650-z PMC9700905 36434667

[171] Pittella, F. et al. Enhanced endosomal escape of siRNA-incorporating hybrid nanoparticles from calcium phosphate and PEG-block charge-conversional polymer for efficient gene knockdown with negligible cytotoxicity. *Biomaterials* **32**, 3106–3114 (2011). 21272932 10.1016/j.biomaterials.2010.12.057

[172] Gu, Y. et al. mRNA delivery enabled by metal–organic nanoparticles. *Nat. Commun.* **15**, 9664 (2024). 39511206 10.1038/s41467-024-53969-w PMC11544223

[173] Jiang, Y., Huo, S., Hardie, J., Liang, X. J. & Rotello, V. M. Progress and perspective of inorganic nanoparticle-based siRNA delivery systems. *Expert Opin. Drug Deliv.* **13**, 547–559 (2016). 26735861 10.1517/17425247.2016.1134486 PMC4914043

[174] Kozielski, K. L., Tzeng, S. Y. & Green, J. J. Bioengineered nanoparticles for siRNA delivery. *Wiley Interdiscip. Rev. Nanomed. Nanobiotechnol.* **5**, 449–468 (2013). 23821336 10.1002/wnan.1233 PMC3972625

[175] Oishi, M., Nakaogami, J., Ishii, T. & Nagasaki, Y. Smart PEGylated Gold nanoparticles for the cytoplasmic delivery of siRNA to induce enhanced gene silencing. *Chem. Lett.* **35**, 1046–1047 (2006).

[176] Artiga, Á., Serrano-Sevilla, I., De Matteis, L., Mitchell, S. G. & de la Fuente, J. M. Current status and future perspectives of gold nanoparticle vectors for siRNA delivery. *J. Mater. Chem. B* **7**, 876–896 (2019). 32255093 10.1039/c8tb02484g

[177] Niu, J. et al. Transdermal gene delivery by functional peptide-conjugated cationic gold nanoparticle reverses the progression and metastasis of cutaneous melanoma. *ACS Appl. Mater. interfaces* **9**, 9388–9401 (2017). 28252938 10.1021/acsami.6b16378

[178] Nor Azlan, A. Y. H., Katas, H., Mohamad Zin, N. & Fauzi, M. B. Dual action gels containing DsiRNA loaded gold nanoparticles: augmenting diabetic wound healing by promoting angiogenesis and inhibiting infection. *Eur. J. Pharm. Biopharm.* **169**, 78–90 (2021). 34582971 10.1016/j.ejpb.2021.09.007

[179] Han, X., Mitchell, M. J. & Nie, G. Nanomaterials for therapeutic RNA delivery. *Matter* **3**, 1948–1975 (2020).

[180] Xia, T. et al. Polyethyleneimine coating enhances the cellular uptake of mesoporous silica nanoparticles and allows safe delivery of siRNA and DNA constructs. *ACS Nano* **3**, 3273–3286 (2009). 19739605 10.1021/nn900918w PMC3900639

[181] Chen, X. et al. Metal-phenolic networks-encapsulated cascade amplification delivery nanoparticles overcoming cancer drug resistance via combined starvation/chemodynamic/chemo therapy. *Chem. Eng. J.* **442**, 136221 (2022).

[182] Lim, C. C., Chia, L. Y. & Kumar, P. V. Dendrimer-based nanocomposites for the production of RNA delivery systems. *OpenNano* **13**, 100173 (2023).

[183] Das, S., Bhavesha, C., Shishira, P. S., Varshini, A. & Biswas, S. Emerging dendrimer-based RNA delivery strategies. *Nanomedicine* **20**, 835–849 (2025). 40178336 10.1080/17435889.2025.2485023 PMC11988225

[184] Chahal, J. S. et al. Dendrimer-RNA nanoparticles generate protective immunity against lethal Ebola, H1N1 influenza, and *Toxoplasma gondii* challenges with a single dose. *Proc. Natl. Acad. Sci. USA* **113**, E4133–E4142 (2016). 27382155 10.1073/pnas.1600299113 PMC4961123

[185] Sonawane, N. D., Szoka, F. C. Jr. & Verkman, A. S. Chloride accumulation and swelling in endosomes enhances DNA transfer by polyamine-DNA polyplexes. *J. Biol. Chem.* **278**, 44826–44831 (2003). 12944394 10.1074/jbc.M308643200

[186] Joubert, F. et al. Precise and systematic end group chemistry modifications on PAMAM and poly(l-lysine) dendrimers to improve cytosolic delivery of mRNA. *J. Controlled Release* **356**, 580–594 (2023). 10.1016/j.jconrel.2023.03.011 36918085

[187] Kisakova, L. A., Apartsin, E. K., Nizolenko, L. F. & Karpenko, L. I. Dendrimer-mediated delivery of DNA and RNA vaccines. *Pharmaceutics* **15**, 1106 (2023). 37111593 10.3390/pharmaceutics15041106 PMC10145063

[188] Li, J. & Loh, X. J. Cyclodextrin-based supramolecular architectures: syntheses, structures, and applications for drug and gene delivery. *Adv. Drug Deliv. Rev.* **60**, 1000–1017 (2008). 18413280 10.1016/j.addr.2008.02.011

[189] Chaturvedi, K. et al. Cyclodextrin-based siRNA delivery nanocarriers: a state-of-the-art review. *Expert Opin. drug Deliv.* **8**, 1455–1468 (2011). 21867463 10.1517/17425247.2011.610790

[190] Hu-Lieskovan, S., Heidel, J. D., Bartlett, D. W., Davis, M. E. & Triche, T. J. Sequence-specific knockdown of EWS-FLI1 by targeted, nonviral delivery of small interfering RNA inhibits tumor growth in a murine model of metastatic Ewing’s sarcoma. *Cancer Res.* **65**, 8984–8992 (2005). 16204072 10.1158/0008-5472.CAN-05-0565

[191] Brahmamdam, P. et al. Targeted delivery of siRNA to cell death proteins in sepsis. *Shock* **32**, 131–139 (2009). 19033888 10.1097/SHK.0b013e318194bcee PMC2950011

[192] Qu, N. et al. Albumin nanoparticle-based drug delivery systems. *Int. J. Nanomed.* **19**, 6945–6980 (2024). 10.2147/IJN.S467876 PMC11246635 39005962

[193] Son, S. et al. Self-crosslinked human serum albumin nanocarriers for systemic delivery of polymerized siRNA to tumors. *Biomaterials* **34**, 9475–9485 (2013). 24050874 10.1016/j.biomaterials.2013.08.085

[194] Yedomon, B., Fessi, H. & Charcosset, C. Preparation of bovine serum albumin (BSA) nanoparticles by desolvation using a membrane contactor: a new tool for large scale production. *Eur. J. Pharm. Biopharm.* **85**, 398–405 (2013). 23811438 10.1016/j.ejpb.2013.06.014

[195] Piao, L. et al. Human serum albumin-coated lipid nanoparticles for delivery of siRNA to breast cancer. *Nanomedicine* **9**, 122–129 (2013). 22542825 10.1016/j.nano.2012.03.008 PMC3605725

[196] Pärnaste, L., Arukuusk, P., Langel, K., Tenson, T. & Langel, Ü. The formation of nanoparticles between small interfering RNA and amphipathic cell-penetrating peptides. *Mol. Ther. Nucleic Acids* **7**, 1–10 (2017). 28624185 10.1016/j.omtn.2017.02.003 PMC5363680

[197] Falato, L., Gestin, M. & Langel, Ü. in *Design and Delivery of SiRNA Therapeutics* (eds Ditzel, H. J. et al.) 329–352 (Springer, 2021).

[198] Yokoo, H., Oba, M. & Uchida, S. Cell-penetrating peptides: emerging tools for mRNA delivery. *Pharmaceutics* **14**, 78 (2022). 10.3390/pharmaceutics14010078 PMC8781296 35056974

[199] Miliotou, A. N. et al. Development of a novel PTD-mediated IVT-mRNA delivery platform for potential protein replacement therapy of metabolic/genetic disorders. *Mol. Ther. Nucleic Acids* **26**, 694–710 (2021). 34703653 10.1016/j.omtn.2021.09.008 PMC8517095

[200] Muskan, M. et al. Therapeutic potential of RNA-enriched extracellular vesicles: the next generation in RNA delivery via biogenic nanoparticles. *Mol. Ther.* **32**, 2939–2949 (2024). 38414242 10.1016/j.ymthe.2024.02.025 PMC11403218

[201] Zeng, H. et al. Current strategies for exosome cargo loading and targeting delivery. *Cells* **12**, 1416 (2023). 10.3390/cells12101416 PMC10216928 37408250

[202] McKenzie, A. J. et al. KRAS-MEK signaling controls Ago2 sorting into exosomes. *Cell Rep.* **15**, 978–987 (2016). 27117408 10.1016/j.celrep.2016.03.085 PMC4857875

[203] Driscoll, J. et al. Using aptamers for targeted delivery of RNA therapies. *Mol. Ther.* **33**, 1344–1367 (2025). 40045577 10.1016/j.ymthe.2025.02.047 PMC11997499

[204] Baek, S. E. et al. RNA aptamer-conjugated liposome as an efficient anticancer drug delivery vehicle targeting cancer cells in vivo. *J. Controlled Release* **196**, 234–242 (2014). 10.1016/j.jconrel.2014.10.018 25450401

[205] Dassie, J. P. et al. Systemic administration of optimized aptamer-siRNA chimeras promotes regression of PSMA-expressing tumors. *Nat. Biotechnol.* **27**, 839–849 (2009). 19701187 10.1038/nbt.1560 PMC2791695

[206] Zhong, R. et al. Hydrogels for RNA delivery. *Nat. Mater.* **22**, 818–831 (2023). 36941391 10.1038/s41563-023-01472-w PMC10330049

[207] Luo, W., Cheng, W., Ni, S., Zhu, X. & Wu, M. A review of stimuli-responsive hydrogels for RNA delivery: from material innovations to clinical barriers. *Int. J. Biol. Macromol.* **318**, 144862 (2025). 40482738 10.1016/j.ijbiomac.2025.144862

[208] Duong, H. T. T. et al. Degradation-regulated architecture of injectable smart hydrogels enhances humoral immune response and potentiates antitumor activity in human lung carcinoma. *Biomaterials* **230**, 119599 (2020). 31718883 10.1016/j.biomaterials.2019.119599

[209] Ding, L. et al. A self-assembled RNA-triple helix hydrogel drug delivery system targeting triple-negative breast cancer. *J. Mater. Chem. B* **8**, 3527–3533 (2020). 31737891 10.1039/c9tb01610d

[210] Bereczki, Z. et al. Mitigating off-target effects of small RNAs: conventional approaches, network theory and artificial intelligence. *Br. J. Pharm.* **182**, 340–379 (2025). 10.1111/bph.17302 39293936

[211] Wang, P., Zhou, Y. & Richards, A. M. Effective tools for RNA-derived therapeutics: siRNA interference or miRNA mimicry. *Theranostics* **11**, 8771–8796 (2021). 34522211 10.7150/thno.62642 PMC8419061

[212] Farina, F. M., Weber, C. & Santovito, D. The emerging landscape of non-conventional RNA functions in atherosclerosis. *Atherosclerosis* **374**, 74–86 (2023). 36725418 10.1016/j.atherosclerosis.2023.01.009

[213] Lu, J. et al. tRNA-derived fragments: unveiling new roles and molecular mechanisms in cancer progression. *Int. J. Cancer* **155**, 1347–1360 (2024). 38867475 10.1002/ijc.35041

[214] Gupta, T., Malkin, M. G. & Huang, S. tRNA function and dysregulation in cancer. *Front. Cell Dev. Biol.* **10**, 886642 (2022). 35721477 10.3389/fcell.2022.886642 PMC9198291

[215] Li, X. et al. tRNA-derived small RNAs: novel regulators of cancer hallmarks and targets of clinical application. *Cell Death Discov.* **7**, 249 (2021). 34537813 10.1038/s41420-021-00647-1 PMC8449783

[216] Statello, L., Guo, C.-J., Chen, L.-L. & Huarte, M. Gene regulation by long non-coding RNAs and its biological functions. *Nat. Rev. Mol. Cell Biol.* **22**, 96–118 (2021). 33353982 10.1038/s41580-020-00315-9 PMC7754182

[217] Liu, J., Ali, M. K. & Mao, Y. Emerging role of long non-coding RNA MALAT1 related signaling pathways in the pathogenesis of lung disease. *Front. Cell Dev. Biol.* **11**, 1149499 (2023). 37250901 10.3389/fcell.2023.1149499 PMC10213921

[218] Gil-Cabrerizo, P., Simon-Yarza, T., Garbayo, E. & Blanco-Prieto, M. J. Navigating the landscape of RNA delivery systems in cardiovascular disease therapeutics. *Adv. Drug Deliv. Rev.* **208**, 115302 (2024). 38574952 10.1016/j.addr.2024.115302

[219] Liang, J. et al. Small nucleolar RNAs: insight into their function in cancer. *Front. Oncol.* **9**, 587 (2019). 31338327 10.3389/fonc.2019.00587 PMC6629867

[220] Wang, Y., Fu, M., Zheng, Z., Feng, J. & Zhang, C. Small nucleolar RNAs: biological functions and diseases. *MedComm* **6**, e70257 (2025). 40584410 10.1002/mco2.70257 PMC12205218

[221] Kristensen, L. S. et al. The biogenesis, biology and characterization of circular RNAs. *Nat. Rev. Genet.* **20**, 675–691 (2019). 31395983 10.1038/s41576-019-0158-7

[222] Zhang, Z. et al. Advances in engineering circular RNA vaccines. *Pathogens* **13**, 692 (2024). 10.3390/pathogens13080692 PMC11356823 39204292

[223] Zhang, L., Liu, J. & Hou, Y. Classification, function, and advances in tsRNA in non-neoplastic diseases. *Cell Death Dis.* **14**, 748 (2023). 37973899 10.1038/s41419-023-06250-9 PMC10654580

[224] Kamola, P. J., Nakano, Y., Takahashi, T., Wilson, P. A. & Ui-Tei, K. The siRNA non-seed region and its target sequences are auxiliary determinants of off-target effects. *PLoS Comput. Biol.* **11**, e1004656 (2015). 26657993 10.1371/journal.pcbi.1004656 PMC4676691

[225] Swayze, E. E. et al. Antisense oligonucleotides containing locked nucleic acid improve potency but cause significant hepatotoxicity in animals. *Nucleic Acids Res.* **35**, 687–700 (2007). 17182632 10.1093/nar/gkl1071 PMC1802611

[226] Chen, Q. X., Wang, W. P., Zeng, S., Urayama, S. & Yu, A. M. A general approach to high-yield biosynthesis of chimeric RNAs bearing various types of functional small RNAs for broad applications. *Nucleic Acids Res*. **43**, 3857–3869 (2015). 25800741 10.1093/nar/gkv228 PMC4402540

[227] Patel, S. et al. Brief update on endocytosis of nanomedicines. *Adv. Drug Deliv. Rev.* **144**, 90–111 (2019). 31419450 10.1016/j.addr.2019.08.004 PMC6986687

[228] Mullard, A. Small molecules against RNA targets attract big backers. *Nat. Rev. Drug Discov.* **16**, 813–815 (2017). 29180732 10.1038/nrd.2017.239

[229] Dhillon, S. Risdiplam: first approval. *Drugs* **80**, 1853–1858 (2020). 33044711 10.1007/s40265-020-01410-z

[230] Perry, C. M. & Balfour, J.A. Fomivirsen. *Drugs* **57**, 375–380 (1999). 10.2165/00003495-199957030-00010 10193689

[231] Blom, D. J., Raal, F. J., Santos, R. D. & Marais, A. D. Lomitapide and mipomersen-inhibiting microsomal triglyceride transfer protein (MTP) and apoB100 synthesis. *Curr. Atheroscler. Rep.* **21**, 48 (2019). 31741187 10.1007/s11883-019-0809-3

[232] Nakamura, A. & Takeda, S. Exon-skipping therapy for Duchenne muscular dystrophy. *Neuropathology* **29**, 494–501 (2009). 19486303 10.1111/j.1440-1789.2009.01028.x

[233] Heo, Y. A. Golodirsen: first approval. *Drugs* **80**, 329–333 (2020). 32026421 10.1007/s40265-020-01267-2

[234] Shirley, M. Casimersen: first approval. *Drugs* **81**, 875–879 (2021). 33861387 10.1007/s40265-021-01512-2

[235] Scott, L. J. Givosiran: first approval. *Drugs* **80**, 335–339 (2020). 32034693 10.1007/s40265-020-01269-0

[236] Scott, L. J. & Keam, S. J. Lumasiran: first approval. *Drugs* **81**, 277–282 (2021). 33405070 10.1007/s40265-020-01463-0

[237] Mendonça, S. A., Lorincz, R., Boucher, P. & Curiel, D. T. Adenoviral vector vaccine platforms in the SARS-CoV-2 pandemic. *NPJ Vaccines* **6**, 97 (2021). 34354082 10.1038/s41541-021-00356-x PMC8342436

[238] Lamb, Y. N. BNT162b2 mRNA COVID-19 vaccine: first approval. *Drugs* **81**, 495–501 (2021). 33683637 10.1007/s40265-021-01480-7 PMC7938284

[239] Li, J. et al. Development of bivalent mRNA vaccines against SARS-CoV-2 variants. *Vaccines* **10**, 1807 (2022). 10.3390/vaccines10111807 PMC9693459 36366316

[240] Wang, Y.-S. et al. mRNA-based vaccines and therapeutics: an in-depth survey of current and upcoming clinical applications. *J. Biomed. Sci.* **30**, 84 (2023). 37805495 10.1186/s12929-023-00977-5 PMC10559634

[241] Bahr-Mahmud, H. et al. Preclinical characterization of an mRNA-encoded anti-Claudin 18.2 antibody. *Oncoimmunology* **12**, 2255041 (2023). 37860278 10.1080/2162402X.2023.2255041 PMC10583639

[242] Essink, B. et al. The safety and immunogenicity of two Zika virus mRNA vaccine candidates in healthy flavivirus baseline seropositive and seronegative adults: the results of two randomised, placebo-controlled, dose-ranging, phase 1 clinical trials. *Lancet Infect. Dis.* **23**, 621–633 (2023). 36682364 10.1016/S1473-3099(22)00764-2

[243] Panther, L. et al. 2892. Safety and immunogenicity of mRNA-1647, an mRNA-based cytomegalovirus vaccine in healthy adults: results of a phase 2, randomized, observer-blind, placebo-controlled, dose-finding trial. *Open Forum Infect. Dis*.**10**,ofad500.2475(2023).

[244] Zhong, L. et al. Urgency and necessity of Epstein-Barr virus prophylactic vaccines. *NPJ Vaccines* **7**, 159 (2022). 36494369 10.1038/s41541-022-00587-6 PMC9734748

[245] Wilson, E. et al. Efficacy and safety of an mRNA-based RSV PreF vaccine in older adults. *N. Engl. J. Med*. **389**, 2233–2244 (2023). 38091530 10.1056/NEJMoa2307079

[246] Pardi, N., Hogan, M. J., Porter, F. W. & Weissman, D.mRNA vaccines—a new era in vaccinology.*Nat. Rev. Drug Discov.* **17**, 261–279 (2018). 29326426 10.1038/nrd.2017.243 PMC5906799

[247] Sydow, E., Mustafa, A. S., Hanif, A., Tunio, J. & Hanif, S. N. M. Recent updates on mRNA vaccines. *Vaccines* **10**, 1209 (2022). 10.3390/vaccines10081209 PMC9416164 36016096

[248] Lee, I. T. et al. Safety and immunogenicity of a phase 1/2 randomized clinical trial of a quadrivalent, mRNA-based seasonal influenza vaccine (mRNA-1010) in healthy adults: interim analysis. *Nat. Commun.* **14**, 3631 (2023). 37336877 10.1038/s41467-023-39376-7 PMC10279702

[249] Philippidis, A. CASGEVY makes history as FDA approves first CRISPR/Cas9 genome edited therapy. *Hum. Gene Ther.* **35**, 1–4 (2024). 38231658 10.1089/hum.2023.29263.bfs

[250] Tao, R. et al. Revolutionizing cancer treatment: enhancing CAR-T cell therapy with CRISPR/Cas9 gene editing technology. *Front. Immunol.* **15**, 1354825 (2024). 38449862 10.3389/fimmu.2024.1354825 PMC10914996

[251] Chehelgerdi, M. et al. Correction: Comprehensive review of CRISPR‑based gene editing: mechanisms, challenges, and applications in cancer therapy. *Mol. Cancer* **23**, 43 (2024). 38195537 10.1186/s12943-023-01925-5 PMC10775503

[252] Kang, C. & Scott, L. J. Voretigene neparvovec: a review in RPE65 mutation-associated inherited retinal dystrophy. *Mol. Diagn. Ther.* **24**, 487–495 (2020). 32535767 10.1007/s40291-020-00475-6

